# Photobiomodulation for the treatment of knee osteoarthritis: therapeutic effects and molecular mechanism

**DOI:** 10.3389/fcell.2026.1744761

**Published:** 2026-03-02

**Authors:** Peng Xia, Tianxiang Fan, Yangxi Huang, Hanwen Zheng, Ruoxi Ma, Wenjin Zhou, Zhi Yao, Deli Wang, Guoqing Cui, Marco Pang, Ye Li, Siu Ngor Fu

**Affiliations:** 1 Department of Rehabilitation Sciences, The Hong Kong Polytechnic University, Hong Kong SAR, China; 2 School of Nursing, LKS Faculty of Medicine, The University of Hong Kong, Hong Kong SAR, China; 3 Department of Bone and Joint Surgery, Peking University Shenzhen Hospital, Shenzhen, Guangdong, China; 4 Department of Rehabilitation, Peking University Third Hospital, Department of Sports Medicine, Peking University Third Hospital, Institute of Sports Medicine of Peking University, Beijing, China

**Keywords:** laser, LED, mechanism, osteoarthritis, photobiomodulation

## Abstract

Knee osteoarthritis (KOA) is a common chronic and degenerative disease, particularly prevalent in the ageing population. The pathological features of KOA include articular cartilage degeneration, osteophyte formation, subchondral bone changes, and synovial hyperplasia. Photobiomodulation (PBM) involves the application of non-ionizing light sources including laser and light-emitting diodes (LED) and broadband light in the visible and near-infrared spectrum to produce stimulatory effect on healing and modulate the inflammatory process in different tissues, including synovium and cartilage of KOA. However, the therapeutic effectiveness and the molecular mechanism of PBM are not fully understood. The results of clinical studies regarding the effects of PBM on KOA are controversial due to differences in study design and execution among these studies. In addition, the lack of unified standards for the optimal treatment strategies, parameters and courses, which has hampered the application of PBM in KOA. In this review, we synthesized clinical and preclinical evidence to evaluate PBM’s efficacy. Our analysis indicates that PBM offers robust symptomatic relief (pain and inflammation) for KOA. However, while preclinical models suggest disease-modifying potential (e.g., cartilage protection), its clinical efficacy in structural regeneration remains speculative and requires further validation through long-term imaging studies.

## Introduction

Knee osteoarthritis (KOA) is the commonest joint disease in ageing population. Its occurrence and development are related to chronic joint injury, ageing, obesity, metabolic bone disease and genetic factors. In clinical practice, knee pain, limited movement, dysfunction, joint deformity and even disability are common symptoms of KOA (Hawker, 2019). According to a study conducted by the Harvard School of Public Health and the World Health Organization, which has been tracking KOA and hip arthritis for nearly 30 years in 204 countries and territories, there are currently an estimated 595 million people with OA worldwide, with 15 million new cases each year (Steinmetz et al., 2023). Another study confirmed that the global prevalence of KOA is 16% in people aged 15 years and older, and 22.9% in people aged 40 years and older. In 2020, an estimated 654.1 million people aged 40 years and older will have KOA worldwide, and 86.7 million people aged 20 years and older will have KOA annually (Cui et al., 2020). Therefore, addressing issues related to patients with KOA is meaningful.

The pathological features of KOA may include articular cartilage degeneration, osteophyte formation, subchondral bone changes, and synovial hyperplasia of the joint (Charlier et al., 2019). At present, the pathogenesis of KOA is still not completely clear. Studies suggest that various cytokines, a variety of metalloproteinases, signalling pathways, chondrocyte senescence, chondrocyte apoptosis and autophagy, estrogen and other aspects may play direct or indirect roles in the development of KOA (Giorgino et al., 2023). Due to the complex pathogenesis of KOA, there are no effective disease-modifying drugs to cure KOA, and conventional drugs still have certain side effects (Cao et al., 2020). Physical therapy is non-invasive, economical and convenient, with few adverse reactions (Wang et al., 2022). Many studies have demonstrated that physical therapies such as electrotherapy, ultrasound, extracorporeal shockwave therapy (ESWT), phototherapy, pulsed electromagnetic field (PEMF), and whole-body vibration (WBV) are beneficial for relieving pain and enhancing activities of daily living in early KOA (Letizia Mauro et al., 2021).

Phototherapy is one of the most commonly used physical therapies with the advantages of fewer side effects, lower cost, and higher safety, involving the use of different spectrums of light to treat diseases and promote the recovery of physical functions. At present, the most studied phototherapy for KOA is photobiomodulation (PBM) (Gendron and Hamblin, 2019). PBM therapy utilizes non-ionizing light sources, including laser and light-emitting diodes (LED) in the visible and near-infrared spectrum (Gendron and Hamblin, 2019). It is confirmed that PBM therapy produces a stimulatory effect on healing and has the ability to modulate the inflammatory process in various tissues, including synovium and cartilage of KOA (Glass, 2021a; Oliveira et al., 2021). Some studies have demonstrated that PBM therapy can relieve pain and joint stiffness, reduce knee swelling, and increase functional performance in KOA patients (Ammar, 2014; Vassão et al., 2020; Alqualo-Costa et al., 2021; Vassão et al., 2021b).

Although the effects of PBM in KOA patients has been studied for many years, the therapeutic effectiveness and the molecular mechanism are not fully understood. The effects of PBM for KOA are controversial due to differences in study design and execution among these studies (Huang et al., 2015; Rayegani et al., 2017; Wyszyńska and Bal-Bocheńska, 2018; Stausholm et al., 2019; Song et al., 2020; Wu et al., 2022). In addition, there are no unified standards for the optimal treatment strategies, parameters and course, which hamper the application of PBM in KOA. In this review, we summarized the studies of different devices of PBM in KOA and explored the therapeutic effects and molecular mechanism involved.

### History, application and mechanism of PBM

The history of phototherapy can be traced back to the early 20th century, initially used for surgery and skin conditions. Niels Finsen was awarded the Nobel Prize in 1903 for his pioneering use of ultraviolet light to treat lupus vulgaris (Roelandts, 2002). In the 1960 s, Mester et al. studied the safety of 694.3 nm low-level laser irradiation on tumor models in mice, finding that it had no adverse effects on skin development and even promoted hair regeneration in shaved areas (Mester et al., 1968). In addition, they found that 14 days laser (1.0 J/cm^2^) irradiation could enhance wound healing in mice and promote the healing of ulcers in diabetic foot patients (Mester et al., 1971; Mester et al., 1976). In 2016, one study officially termed this phenomenon as PBM (Ranjbar and Takhtfooladi, 2016). This phenomenon is a cascade of physiological events induced by the nonthermal exposure of tissue to light at the near-infrared end of the visible spectrum (Glass, 2021a). Currently, PBM therapy devices emit visible red and/or near-infrared light within the spectrum and mainly utilize laser or LED with specific properties including wavelength, total optical power, spot size, power density, pulse structure and exposure duration (Gendron and Hamblin, 2019). Laser is already widely used and the light is monochromatic, coherent, collimated and polarized (Glass, 2021a). LED is a new technology and the light is near monochromatic but neither coherent, collimated, nor polarized. Moreover, LED is cheaper than laser, and it is easy to design as wearable device with different wavelengths (Heiskanen and Hamblin, 2018; Glass, 2021a).

After decades of development, PBM therapy has been widely applied in clinical practice (Glass, 2021b; Bunch, 2023). PBM can directly target the treatment area through light irradiation, while drugs need to be transported through the bloodstream to specific sites for efficacy. This feature enables PBM to be better than conventional drug treatments for some diseases involving poor blood circulation. The mild side effects of PBM therapy include skin irritation, itching, and redness, which are not harmful to the body and do not cause excessive heating of the target tissue (Zamani et al., 2020). Many studies have demonstrated the effects of PBM on musculoskeletal disorders (such as rotator cuff disease (Page et al., 2016), tendinopathy (Tumilty et al., 2010), low-back pain (Glazov et al., 2016), adhesive capsulitis (Page et al., 2014), carpal tunnel syndrome (Rankin et al., 2017), rheumatoid arthritis (Brosseau et al., 2005) and OA (Rayegani et al., 2017)), neurological disorders (such as peripheral nerve injury (Rosso et al., 2018),Alzheimer’s disease (Su et al., 2023), Parkinson’s disease (Foo et al., 2020), spinal cord injury (Ramezani et al., 2020) and retinal protection and regeneration (Valter et al., 2024)), skin conditions (such as acne vulgaris (Fl et al., 2009), androgenic alopecia (Kh et al., 2019) and wound healing (Ozcelik et al., 2008; Dias et al., 2015)) and other diseases (such as chronic obstructive pulmonary disease (Ys et al., 2023), chronic kidney disease (Bian et al., 2022), temporomandibular disorders (Regulski et al., 2023) and oral mucositis (Kauark-Fontes et al., 2023)).

**FIGURE 1:**
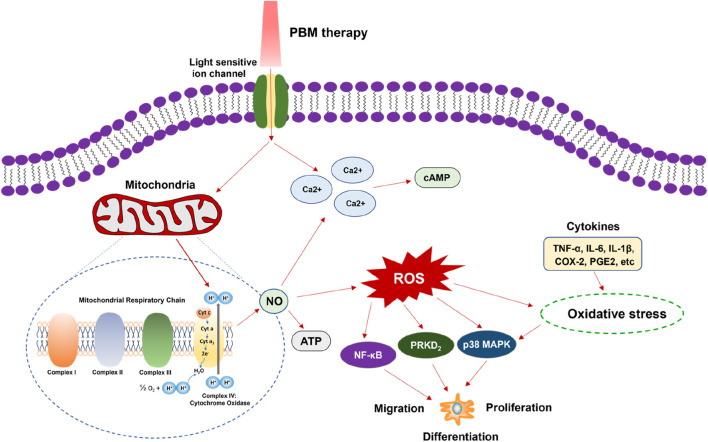
Intracellular mechanism of PBM therapy. The light from PBM therapy device activate photosensitive and can be absorbed by cytochrome c oxidase, located in the fourth complex of the mitochondrial respiratory chain, displacing NO and activating the enzyme. This process increases the production levels of Ca2^+^, ROS, and ATP. Ca2^+^ can interact with ROS and cAMP, and the increase of ROS level results in the activation of NF-κB, p38 MAPK and PRKD2, which related to cell differentiation, proliferation and migration. Oxidative stress caused by elevated ROS can also stimulate p38 MAPK by the presence of cytokines. PBM, photobiomodulation; NO, nitric oxide; ROS, reactive oxygen species; ATP, adenosine triphosphate; cAMP, cyclic adenosine monophosphate; NF-κB, nuclear factor-kappa B; MAPK, mitogen-activated protein kinases; PKD2, protein kinase D2; IL-1β, interleukin 1β; IL-6, interleukin 6; ΤΝF-α, tumor necrosis factor-α; COX-2, cyclooxygenase-2; PGE2, prostaglandin E2.

Although PBM therapy has been used in many diseases, elucidating the mechanism will help us better understand the beneficial effects of PBM on the healing of disease-related cells and tissues, and also help us further improve the PBM devices. Several studies demonstrated the mechanism of PBM is mainly related to mitochondrial respiration, gene expression, cell signaling, growth factor synthesis and inflammation regulation (Karu, 1999; Chung et al., 2012; de Freitas and Hamblin, 2016; Wang et al., 2017). The theory suggests that the light from PBM therapy device activate photosensitive chromophores and can be absorbed by cytochrome c oxidase, located in the fourth complex of the mitochondrial respiratory chain, displacing nitric oxide (NO) and activating the enzyme, thereby leading to a proton gradient. This process increases the production levels of Ca2^+^, reactive oxygen species (ROS), and adenosine triphosphate (ATP). Moreover, Ca2^+^ can interact with ROS and *cyclic* adenosine monophosphate (cAMP), and the increase of ROS level results in the activation of various signaling pathways such as nuclear factor-kappa B (NF-κB), p38 mitogen-activated protein kinases (MAPK) and protein kinase D2 (PRKD2), which related to cell differentiation, proliferation and migration (Dompe et al., 2020; Hennessy and Hamblin, 2017). In addition, oxidative stress caused by elevated ROS can also stimulate p38 MAPK by the presence of cytokines (such as interleukin 1β(IL-1β), IL-6, tumor necrosis factor-α (ΤΝF-α), cyclooxygenase-2 (COX-2), prostaglandin E2 (PGE2), etc.) (Son et al., 2017; Son et al., 2014) (Figure 1).

The recent evidences confirmed that PBM can stimulate healing, promote nerve regeneration, relieve pain, reduce inflammation and improve microcirculation. However, the light source, wavelength, energy density, and duration of light exposure all impact the effectiveness of PBM. Different parameters may have different therapeutic effects on diseases, and they may also yield adverse effects. Although, PBM is believed to promote cell migration and vitality, collagen production, ATP production, increase growth factors and mitochondrial number, enhance gene regulation and inhibit inflammatoion (Karu, 1999; Hamblin, 2017; Glass, 2021a), but the exact mechanisms of PBM therapy in certain diseases are not well understood. Therefore, we need to continue exploring reasonable treatment strategies and the specific mechanism of PBM in disease treatment. In the following content, we primarily conducted an in-depth discussion of PBM therapy for KOA.

### Effectiveness of PBM therapy for KOA patients

To synthesize clinical evidence, we conducted a systematic search of PubMed from January 1992 to December 2023 using keywords “Photobiomodulation,” “laser,” “LED,” “knee,” and “osteoarthritis.” Inclusion criteria: high-level evidence such as randomized controlled trials (RCTs) or systematic reviews (SRs) with/without meta-analysis; human studies on PBM for KOA; English language; focus on therapeutic effects (e.g., pain, function). Exclusion criteria: case reports, observational studies, animal/*in vitro* studies, non-KOA OA, or non-PBM interventions. This yielded 59 studies (primarily RCTs and SRs), summarized in Supplementary Table 1; Table 1, which compares protocols (wavelength, power, energy density, duration, frequency) and outcomes for structured synthesis. Methodological quality of the included RCTs was assessed using the Physiotherapy Evidence Database (PEDro) scale (total score out of 10) (Maher et al., 2003). PEDro scores ranged from 5 to 10, with a mean score of 6.9, indicating overall medium to high methodological quality. Higher-quality studies (PEDro ≥7) tended to report more consistent short-term benefits in pain and function when appropriate parameters and exercise combinations were used.

**TABLE 1:** Summary of clinical studies on PBM therapy for KOA patients.

Category	Subcategory	No. of studies (RCT/SR)	Common wavelengths (nm)	Common energy density (J/cm^2^)	Common total energy per session (J)	Treatment frequency & duration	Primary outcomes	Key findings
**LLLT Alone**	Pure LLLT vs. Placebo	10 RCTs +3 SRs	633–905 (often 830, 904)	0.76–50	5.76–27	2–5 sessions/week, 3–8 weeks (8–20 sessions)	Pain (VAS/WOMAC), function, ROM	Mixed: Many show pain reduction; some ineffective
**LLLT – Laser Acupuncture**	vs. Placebo	9 RCTs +1 SR	650–10,600 (often 780–830 + CO_2_)	4–70	0.48–216	3–5 sessions/week, 4–5 weeks	Pain (VAS), function, biomarkers	Consistent short-term pain reduction and functional improvement
**LLLT + Exercise**	vs. Exercise alone or Placebo + Exercise	11 RCTs +2 SRs	808–904 (also 850, 880)	3–200	2–56	2–3 sessions/week, 3–8 weeks (10–24 sessions)	Pain (VAS/WOMAC), function, muscle strength	Generally superior to exercise alone. Some SRs show controversy
**LLLT – Comparisons**	vs. NES, Ozone, PEMFetc.	6 RCTs	810–980	0.2–91	24–42	2–3 sessions/week, 4–8 weeks	Pain, function, ultrasonography	Similar to NES; less effective than ozone or PEMF.
**HILT Alone or Comparisons**	vs. Placebo/Other therapies	12 RCTs +4 SRs	808–1,064 (often 1,064)	1.5–120	79.2–3,000	1–5 sessions/week, 2–6 weeks (7–20 sessions)	Pain (VAS/WOMAC), function, ROM	Consistently reduces pain; often superior to LLLT or other modalities
**LED**	Alone or vs. LLLT + Exercise	4 RCTs	640–905	0.12–83.2	23.5–1,402	2–3 sessions/week, 2–6 weeks	Pain, function, QOL	Mixed: No benefit alone in one study; comparable to LLLT with exercise

RCT, randomized controlled trial; SR, systematic review; PBM, photobiomodulation; LLLT, Low-Level Laser Therapy; HILT, High-Intensity Laser Therapy; LED, light emitting diodes; VAS, visual analogue scale; ROM, range of motion; WOMAC, Western Ontario and McMaster Universities Osteoarthritis Index; QOL, quality of life; NES, neuromuscular electrical stimulation; PEMF, pulsed electromagnetic field; CO_2_, Carbon Dioxide (for CO_2_ laser in moxibustion).

#### Low-level laser therapy (LLLT)

LLLT is the most studied and widely used PBM therapy, and it can generate energy output up to 500 mW by concentrating laser radiation, resulting in biological stimulation and anti-inflammatory effects. This type of PBM therapy can penetrate the superficial tissue layer with a maximum penetration depth of 2 cm, but it does not produce any thermal sensation during the treatment process (Elvir-Lazo et al., 2020). In the earliest study conducted in 1992, Stelian et al. (1992) provided red (5.1 J/cm^2^, 633 nm) or infrared (5.6 J/cm^2^, 830 nm) LLLT to patients with KOA. After treatment twice a day for 10 days, the results showed that both red and infrared LLLT could alleviate pain and disability, and with no significant difference between the two light emitters. Hegedus et al. (2009) administered 830 nm LLLT to mild or moderate KOA patients twice a week over a 4-week period and measured microcirculatory changes using thermography. They found that 2 months after completing LLLT, a significant improvement was confirmed in the joint pain, circumference, pressure sensitivity, flexion and microcirculation. Jankaew et al. (2023) found that 660 nm or 808 nm LLLT improves muscle strength and functional performance compared to the sham LED in KOA patients. Fukuda et al. (2011) also demonstrated that 9 sessions treatment with 904 nm LLLT improves pain and function over the short term in KOA patients (Gopal et al., 2017). evaluated the behavioral and biochemical effects of LLLT and indicated that 905 nm LLLT represents an effective treatment for short-term improvement in pain, joint space width and other biochemical variables (collagen type-II C telopeptide (CTX-II), matrix metalloproteinase (MMP)-3, MMP-8, MMP-13) in patients with KOA. A systematic review and meta-analysis in 2017 included 14 RCTs, and the results indicated that LLLT seems to be effective in reducing pain and improving function, but had no significant impact on range of motion compared to placebo in KOA patients (Rayegani et al., 2017). Another study reviewed 22 RCTs showed that the pain and disability of KOA were reduced by LLLT at 4–8 J with 785–860 nm wavelength and at 1–3 J with 904 nm wavelength per treatment spot (Stausholm et al., 2019).

Contrary to the above studies, Bülow et al.'s study showed that compared to placebo, there was no significant change in pain and medicine requirements in KOA patients after 9 sessions of 830 nm LLLT (1.5–4.5J/per painful point) treatment (Bülow et al., 1994). In another study, two different dosages (1.5J or 3J/per painful point) of 830 nm LLLT were used to treat KOA patients, and the results indicated that LLLT did not improve pain and physical function after 10 treatment sessions or at the follow-up (Tascioglu et al., 2004). In addition, a systematic review and meta-analysis published in 2015 included 9 RCTs with 518 KOA patients, and demonstrated no therapeutic benefit of LLLT compared with placebo in pain relief and functional improvement, neither immediately post-treatment nor at the 12-week follow-up (Huang et al., 2015). Our opinion is that the failure in pain and function improvement may be related to the laser modality, dosages, treatment duration and wavelength selection.

In order to improve the effectiveness of LLLT on KOA, some studies used a safe technique of laser acupuncture which combined modern laser, traditional acupuncture and moxibustion (Whittaker, 2004). The earliest study included grades 2–3 primary KOA patients and administered 904 nm LLLT for 120-s treatment time on the medial side of the knee to the acupuncture point SP9. They found that laser acupuncture was more effective only in reducing periarticular swelling compared to placebo (Yurtkuran et al., 2007). Multiple studies used LLLT (wavelength 780–830 nm) to intervene in patients with KOA at various acupuncture points, and the results showed that laser acupuncture significantly increased serum beta-endorphin, decreased substance P, and improved pain and disability (Al Rashoud et al., 2014; Helianthi et al., 2016; Mohammed et al., 2018; Liao et al., 2020). Moreover, four studies from the same team demonstrated that combined laser moxibustion (10.6 μm CO_2_ laser which mimics moxibustion) and 650 nm LLLT at acupuncture point Dubi or Ashi had obvious analgesic and function improvement effects in KOA patients compared with the placebo laser (Shen et al., 2009; Zhao et al., 2010; 2021; Lin et al., 2020). A systematic review and meta-analysis by Chen et al. included 7 RCTs indicating that laser acupuncture had significant analgesic effect in KOA in the short term, however, the effect disappeared during the long-term follow-up period (Chen et al., 2019). In the future, it is necessary to standardize the parameters of LLLT and use appropriate acupuncture points to improve the long-term therapeutic effect of laser acupuncture on KOA.

Exercise is one of the most common physical therapies, and it is proven to be effective in pain reduction and function improvement for KOA (Varongot-Reille et al., 2023). Many studies explored the combined effects of LLLT and exercise in patients with KOA. de Matos Brunelli Braghin et al. demonstrated that 15 sessions exercises or exercises combined with 808 nm LLLT significantly improved pain and function compared to the control or LLLT alone, and exercises plus LLLT provided the best results for the gait variables (de Matos Brunelli Braghin et al., 2019). Kholvadia et al. found no significant difference in the reduction of Western Ontario and McMaster Universities osteoarthritis index (WOMAC) pain and functionality scale and knee circumference scores between LLLT and 12 sessions of exercises, but the combination of LLLT and exercise had better outcomes in terms of pain and functional improvement than either LLLT or exercise alone (Kholvadia et al., 2019). A recent study by Robbins et al. demonstrated that while 904 nm LLLT and stretching exercises (24 sessions) yielded comparable therapeutic outcomes when applied independently, their combined application was significantly superior in alleviating resting pain and improving functional measures, including activities of daily living, joint stiffness, muscle shortening, and range of motion (Robbins et al., 2022). In addition, seven RCTs have demonstrated that the addition of LLLT (wavelength 850, 880 or 904 nm) to exercise is more effective than exercise alone for KOA patients in reducing pain, disability, and pain medication usage, and these effects can be maintained for 6 months (Gur et al., 2003; Alfredo et al., 2012; Alfredo et al., 2018; Alfredo et al., 2022; Alghadir et al., 2014; Youssef et al., 2016; Stausholm et al., 2022).

Nevertheless, two RCTs by Vassão et al. found no significant additive effect of PBM when combined with a physical exercise program (Vassão et al., 2020; Vassão et al., 2021a). A deeper analysis of the dosimetric data provides a substantive explanation for these findings. In both studies, a cluster device (808 nm) delivered a total energy of 56 J per knee. Although the total energy was relatively high, it was distributed via a cluster probe with multiple diodes (100 mW each), resulting in a lower peak power and intensity at the targeted deep tissues. In contrast, studies demonstrating a significant additive effect, such as Alfredo et al. (2012) utilized a 904 nm super-pulsed laser. The 904 nm wavelength offers superior tissue penetration compared to 808 nm, and the high peak power of super-pulsed delivery (even at 3 J per point and 6 J/cm^2^) is more effective at reaching the intra-articular environment of the knee. Furthermore, the exercise program implemented by Vassão et al. was highly intensive (including strength training 2 times/week), which might have induced a ‘ceiling effect’. In such cases, if the PBM parameters, particularly wavelength and penetration depth,do not reach a certain therapeutic threshold to modulate the inflammatory microenvironment beyond what exercise already achieves, its incremental benefits remain undetectable. This suggests that the ‘negative’ outcomes were likely due to insufficient photon density at depth and the high efficacy of the exercise intervention itself.

Vassão et al. also reviewed 7 RCTs (including 5 LLLT and 2 high-intensity laser therapy (HILT) studies), and concluded that the effects of PBM association with exercise for pain and functional improvement in KOA remain controversial (Vassão et al., 2021b). A recent systematic and meta-analysis including 14 RCTs revealed that LLLT plus exercise is more effective in reducing pain, but no more effective in improving range of motion, muscle strength and function than placebo LLLT plus exercise in KOA (Malik et al., 2023). We think that these negative results are mainly due to the type of PBM, parameters, dose, treatment sessions and exercise protocols. Therefore, further research is needed to investigate the impact of these factors on the efficacy of PBM combined with exercise in the treatment of KOA.

Some studies have compared the effects of LLLT and other physical therapies, as well as their combined treatment in KOA patients. Melo Mde et al. found that LLLT, neuromuscular electrical stimulation (NES) and combined treatments for KOA patients presented similar improvements in pain, function, strength, electrical activity of the quadriceps and health status. Additionally, only treatments including NES increased muscle mass, and 16 sessions of 810 nm LLLT did not enhance the therapeutic effects of NES on the above evaluated parameters (Melo Mde et al., 2015; de Oliveira Melo et al., 2016). Fakhari et al. demonstrated that both LLLT and ozone therapy significantly decreased pain and improved WOMAC score and the range of joint motion, but the ozone therapy was more effective compared to 980 nm LLLT (Fakhari et al., 2021). Alqualo-Costa, et al. analyzed the effects of interferential current (IFC), LLLT and their combination on pain in KOA patients. They found that LLLT significantly reduced pain at rest than IFC at 6 months follow-up, and LLLT plus IFC were more effective in pain reduction compared to placebo and IFC alone at all time points (Alqualo-Costa et al., 2021). Elboim-Gabyzon et al. claimed that both PEMF therapy and LLLT had positive effects on pain and physical function in patients with grades 2–3 primary KOA, and PEMF was more effective in reducing pain and improving physical function than LLLT (Elboim-Gabyzon and Nahhas, 2023). Paolillo et al. did not study the efficacy of LLLT versus ultrasound, but explored their combined effects. They suggested that 3 months of treatment of LLLT plus ultrasound with or without exercise significantly reduced pain and improved functional performance in women with KOA compared to placebo (Paolillo et al., 2018). Due to the small sample size and the controversy over the treatment parameters of LLLT, further research is needed to compare LLLT with these therapies and their combined effects. In addition, there is no research to compare LLLT with ultrasound, ESWT, short wave, and traditional Chinese medicine treatment (such as acupuncture and moxibustion) in KOA treatment, which is the direction that needs further exploration in the future.

#### High-intensity laser therapy (HILT)

In contrast to LLLT, HILT utilizes scattered laser radiation technology to generate an energy output of >500 mW, enabling deeper tissue penetration (up to 15) and inducing surface hyperthermia (photothermal effect) for effective oxidative reactions and increased ATP production (Elvir-Lazo et al., 2020). The study conducted by Angelova et al. demonstrated that 7 sessions of HILT (wavelength 1,064 nm, power 12W, energy 300/3000J) could be recommended as a treatment for pain relief and function improvement in KOA patients compared with placebo (Angelova and Ilieva, 2016). Akaltun et al. indicated that 10 sessions of HILT combined with exercise was more effective in reducing pain and WOMAC scores, increasing range of motion (ROM) and femur cartilage thickness than placebo laser combined with exercise (Akaltun et al., 2021). Wyszynska et al. conducted the first systematic review of the effect of HILT on KOA based on six studies and found that HILT is an effective treatment for pain reduction and improved function. However, due to the high degree of heterogeneity among the included studies, no meta-analysis was performed (Wyszyńska and Bal-Bocheńska, 2018). Song et al. performed a systematic review and meta-analysis of 6 RCTs to investigate the effects of HILT on KOA patients, and the results showed that HILT significantly decreased pain and improved WOMAC stiffness and function (Song et al., 2020).

Since the therapeutic effect of LLLT in KOA has been confirmed by many studies, several studies have compared the effects of HILT and LLLT on KOA patients. Gworys et al. compared LLLT (810 nm, 400 mW) and HILT (808/905 nm, 1,100 mW) in KOA patients. Although the total energy doses were comparable, the two modalities differ in power density. While LLLT triggers photochemical effects in superficial tissues, the significantly higher peak power of HILT enables deeper photon penetration into the joint capsule and subchondral bone. Consequently, although all methods improved pain and function after 10 sessions, HILT demonstrated superior clinical efficacy due to its greater intensity and penetration depth (Gworys et al., 2012). Kheshie et al. found that the combination of HILT and LLLT with exercise yielded positive outcomes in reducing pain and improving function after a 12 sessions treatment period. Notably, HILT combined with exercises exhibited superior efficacy compared to LLLT combined with exercises, while both treatment modalities outperformed exercise alone in managing patients with KOA (Kheshie et al., 2014). A systematic review and meta-analysis of 10 high-quality RCTs confirmed that adding laser therapy to rehabilitation exercise enhances clinical outcomes in KOA. Notably, the study utilized a network-based indirect comparison to address the lack of direct head-to-head trials. The results highlighted that HILT as an adjunct to exercise yielded superior results in pain reduction and functional recovery compared to LLLT. However, the authors emphasized the need for future direct comparative investigations to consolidate these findings (Ahmad et al., 2022). Since this result of meta-analysis is based on indirect comparison, direct comparison of the two types of laser therapy should be conducted in the future.

Some studies investigated the effects of HILT versus other physical therapies in KOA patients. Mostafa et al. evaluated and compared the effects of HILT and ESWT on KOA patients, and indicated that HILT is more effective than ESWT in KOA for pain, physical function and disability (Mostafa et al., 2022). The study conducted by Samaan et al. demonstrated that the combination of HILT with exercises showed superior outcomes in terms of pain reduction, knee ROM, proprioceptive accuracy, and functional disability compared to the combination of low-intensity pulsed ultrasound with exercises, as well as exercise alone (Samaan et al., 2022). Nazari et al. found that HILT combined with exercise was more effective than conservative physical therapy (CPT) (including transcutaneous electric nerve stimulation (TENS) and ultrasound) combined with exercise and exercise alone in improving pain and function of KOA patients (Nazari et al., 2019). A network meta-analysis included 9 RCTs suggested that HILT may be more effective than other physical therapies (such as LLLT, TENS plus ultrasound, ESWT, thermotherapy plus interferential current) for improving pain and function in KOA patients, however, it may not be clinically effective for improving stiffness (Wu et al., 2022). Due to the limited sample size in the above studies, further large-scale investigations are required to establish a conclusive comparison of the efficacy of HILT with ESWT, ultrasound, TENS, thermotherapy, and interferential current in KOA. Moreover, the comparative effectiveness of HILT with other physical modalities such as short wave, NES and PEMF remains unknown and warrants future investigation.

Other studies have explored the effects of HILT combined with other treatments in KOA. Kim et al. randomly allocated patients with KOA to receive either CPT or CPT combined with HILT. The CPT protocol included hot pack treatment, interferential current therapy, and deep heat diathermy using ultrasonic waves. The results demonstrated that 12 sessions of HILT following CPT significantly improved pain and function compared to CPT alone (Kim et al., 2016). Siriratna et al. also found that HILT combined with CPT (including education on KOA, such as weight reduction, exercise, and lifestyle modification) significantly alleviated pain compared with placebo combined with CPT (Siriratna et al., 2022). Ammendolia et al. demonstrated the efficacy and safety of HILT in treating KOA. Furthermore, when combined with glucosamine/chondroitin sulfate (GS/CS), it can achieve a sustained therapeutic effect for up to 6 months post-treatment (Ammendolia et al., 2021). Alayat et al. suggested that HILT combined with GS and exercise effectively reduced pain WOMAC subscales, synovial thickness compared to the groups receiving GS plus exercise and placebo plus exercise, with no significant differences were observed in medial and lateral femoral cartilage thickness among the three treatment protocols (Alayat et al., 2017). In clinical practice, KOA patients are more likely to be treated with multiple treatments, so more studies are needed to further explore the optimal combination protocol of HILT and other therapies.

#### LED

LED is a sophisticated semiconductor that converts electrical current into incoherent narrow-spectrum light, with wavelengths ranging from ultraviolet to visible and near-infrared bandwidths (247–1,300 nm). This therapeutic approach has garnered attention due to its low cost, noninvasiveness, minimal contraindications, and rare adverse effects (Barolet, 2008). LED-based therapeutics fall under the class II category as classified by the Food and Drug Administration (FDA), requiring clearance based on similarity to existing devices rather than approval necessitating evidence of efficacy and safety. In contrast, traditional medical laser belongs to FDA class III or IV, and it is expensive, potentially hazardous if mishandled, and subject to regulatory restrictions on usage (Glass, 2021a). Therefore, LED has greater commercial appeal than traditional medical lasers.

At present, there are few studies on LED in KOA. Monochromatic infrared energy (MIRE), a kind of LED using an Anodyne Therapy System (model 480; Anodyne Therapy, LLC, Tampa, FL). This device consists of a base power unit and 8 therapy pads, each containing 60 superluminous gallium-aluminum arsenide diodes and delivering a single wavelength of near-infrared photo energy at 890 nm (Burke, 2003). In 2012, Hsieh et al. examined the short-term therapeutic effects of MIRE on patients with KOA. They found no significant differences in the scores of Knee injury and Osteoarthritis Outcome Score (KOOS), Lysholm Knee Scale, Hospital Anxiety and Depression Scale, Multidimensional Fatigue Inventory, Chronic Pain Grade questionnaire, World Health Organization Quality of Life-brief version, and OA Quality of Life Questionnaire after 40 min of MIRE treatment (3 times per week for a total of 6 sessions) compared to the placebo. The results suggested that short-term LED therapy has no beneficial effects on pain, functions, activities, participation, and quality of life in KOA (Hsieh et al., 2012). Ammar et al. investigated the effects of MIRE (treatment time 30 min) plus exercises and LLLT (wavelength 850 nm, treatment time 10 min) plus exercises in KOA patients and suggested that they both have positive effects in improving pain and function after 12 sessions (2 sessions per week for 6 weeks) treatment, however, no significant differences were found between these two modalities (Ammar, 2014). De Paula Gomes et al. used an LED cluster (one 905 nm super-pulsed diode laser, four 875 nm LED and four 640 nm LED) and found that 10 sessions of LED combined with exercises significantly reduced pain in KOA patients compared to exercise alone or exercise plus placebo LED in a short-term protocol (de Paula Gomes et al., 2018). A recent study by Pinto et al. used LED (Light-Aid system with a 100 LED divided into 4 LED clusters with 25 LED each, wavelength 850 nm) to intervene in KOA patients for 10 sessions, and report significant pain relief in a short- and medium-term after LED treatment when compared to placebo (Pinto et al., 2022).

In general, research on LED therapy for KOA has not received enough attention. The effects of LED in KOA still need to be confirmed by more clinical studies, and the device selection and parameters of LED still need to be further studied. Moreover, the comparison of the effectiveness of the LED with other therapies, as well as the combined use of LED and other therapies, and the development of more convenient and effective wearable LED devices are all future LED research directions in KOA.

### The molecular mechanism of PBM for KOA

**FIGURE 2:**
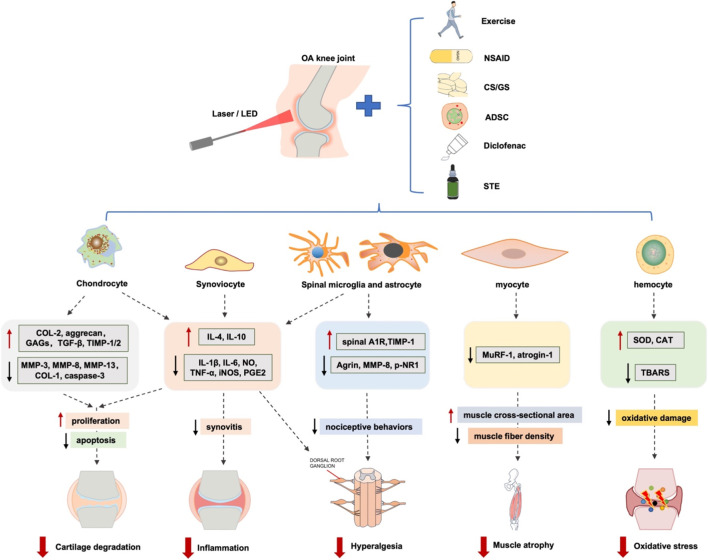
Molecular biology of LED and combined therapies to KOA. PBM alone or combined with exercise, NSAID, CS, GS, ADSC and STE affect the gene expression in KOA related cells such as chondrocytes, synoviocytes, spinal microglia and astrocyte, myocyte and hemocyte, and then decrease cartilage degeneration, inflammation, hyperalgesia, muscle atrophy and oxidative stress. LED, light emitting diode; KOA, knee osteoarthritis; NSAID, steroid anti-inflammatory drug; CS, chondroitin sulfate; GS, glucosamine sulfate; ADSC, adipose-derived stromal cell; STE, D iclofenac, S. Tuberculata extract.

Several *in vivo* and *in vitro* studies have explored the effects of PBM on KOA and attempted to elucidate the underlying mechanisms that produce these effects. These studies mainly confirmed the effectiveness of PBM in cells or animal models of KOA, focusing on inflammation, cartilage degeneration, nociception, hyperalgesia, muscle atrophy and oxidative stress damage. We searched and included these studies, with details described below (Table 2; Figure 2).

**TABLE 2:** Molecular evidences of PBM for KOA.

Reference	Injury model	Animal	Combined treatment	Parameters	Duration of treatment	Outcome measure	Results
Cartilage degeneration							
Lin et al. (2012)	ACLT	Rabbit	-	λ(nm): 810 P (mW): 0–1,000 Beam area (cm^2^): 0.25 T (min):10 ED (J/cm^2^): 3 E(J):N/A PD (mW/cm^2^):5	5 days/week 2 weeks	HA, IHC, TUNEL	↓ the cartilage disorganization ↓ caspase-3 (cartilage) - caspase-8 and chondrocyte apoptosis
Oliveira et al. (2013)	ACLT	Rat	-	λ(nm): 830 P (mW):30 Beam area (cm^2^): 0.028 T(s):10/47/points, 2 points ED (J/cm^2^): 10/50 E(J):0.3/1.4 PD (mW/cm^2^): N/A	5 days/interval of 2 days 3/6 weeks	HA, IHC	↓ number of chondrocytes (10 J/cm^2^) ↓ COL-1 (50 J/cm^2^) (cartilage) - cartilage thickness - Mankin score - IL-1β, TNF-α, MMP-13
Moon et al. (2014)	ACLT	Rat	-	λ(nm): 850 P (mW):1/2/5 Beam area (cm^2^): 0.0451 T (min):1 ED (J/cm^2^): 1.33/2.66/6.65 E(J):N/A PD (mW/cm^2^):N/A	1/day 7 days 4 weeks	HA, ultraviolet spectrophotometric assay, physical examination, BrdU assay	↑ functional movement of knee joint ↓ edematous changes ↑ GAGs (cartilage) ↑ chondrocyte proliferation ↓ cartilage degradation
Oshima et al. (2011)	ACLT	Rabbit	-	λ(nm):630/870 P (mW):N/A Beam area (cm^2^): 44 T (min):10 ED (J/cm^2^):2/2.5 E(J):88/110 PD (mW/cm^2^):3.2/4	5 days/week 5 weeks	HA, RT-PCR	↑ cartilage regeneration ↑ COL-2 (cartilage) ↓ TNF-α(synovial membrane) - aggrecan
Trevisan et al. (2020)	ACLT	Rat	-	λ(nm):850 P (mW):200 Beam area (cm^2^):0.5 T(s):30/point, 2 points ED (J/cm^2^):N/A E(J):6 PD (mW/cm^2^):0.4	3 days/week 4 weeks	HA, gate analysis, IHC	- gate analysis ↓ irregularities along fibrillation and the joint tissue ↑ COL-2, TGF-β(cartilage)
**Inflammation**							
Wang et al. (2014)	ACLT	Rabbit	-	λ(nm): 830 P (mW):50 Beam area (cm^2^): 0.028 T (min):5/points, 6 points ED (J/cm^2^): 4.8 E(J):0.13 PD (mW/cm^2^):N/A	3/week 2/4/6/8 weeks	Behavior test, HA, RT-PCR	6 weeks ↓ knee pain and synovium inflammation ↓ cartilage damage of medical femoral condyle ↓ IL-1β, iNOS, MMP-3 (cartilage) ↑ TIMP-1 8 weeks ↓ cartilage damage of medical and lateral femoral condyles and medical tibial plateau ↑ COL-2, aggrecan, TGF-β
Tim et al. (2022)	ACLT	Rat	-	λ(nm): 808 P (mW):50 Beam area (cm^2^): 0.028 T(s):16/28 ED (J/cm^2^): 28/50 E(J):0.8/1.4 PD (mW/cm^2^):1.78	*In vitro* Every 24 h for 1/3/5 days *In vivo* 3 days/week 4/8 weeks treatment	HA, IHC, RT-PCR	↑ IL-4, IL-10, COL-2, aggrecan, TGF-β (cartilage) ↓ IL-1β ↑ chondrocyte proliferation
Yamada et al. (2020)	MIA	Rat	-	λ(nm): 904 P (mW): N/A Beam area (cm^2^): N/A T(s): N/A ED (J/cm^2^): 6/18 E(J):1/point PD (mW/cm^2^):N/A	3 days/week 8 sessions	Biochemical analyses, pain evaluation, ELISA	↓ the activity of neutrophils ↓ pain ↓ NO and TNF-α, IL-1β, IL-6 ((knee structures:cruciate and collaterals ligaments, capsule, meniscus)
da Rosa et al. (2012)	Papain injection	Rat	-	λ(nm): 808/660 P (mW): 100 Beam area (cm^2^): 0.028 T(s): 40/points, 4 points ED (J/cm^2^): 142 E(J): 4 PD (W/cm^2^): 3.57	7/14/21 days	HA	808 nm LLLT ↑ angiogenesis ↓ inflammatory exudation ↓ cartilage fibrosis
Tomazoni et al. (2017)	Papain injection	Rat	Exercise NSAID	λ(nm): 830 P (mW):100 Beam area (cm^2^): 0.028 T (min):1 ED (J/cm^2^): 214.2 E(J):6 PD (W/cm^2^):3.57	3 days/week, 8 weeks	RT-PCR, ELISA	↓ IL-1β, IL-6, TNF-α(cartilage) ↓ PGE2(blood plasma)
Tomazoni et al. (2016)	Papain injection	Rat	Exercise, Diclofenac NSAID	λ(nm): 830 P (mW):100 Beam area (cm^2^): 0.028 T (min):1 ED (J/cm^2^):214.2 E(J):6 PD (W/cm^2^):3.57	3 days/week, 8 weeks	HA, Total cell count, MPO activity analysis, RT-PCR	↓ morphological alterations ↓ the total number of cells in the inflammatory infiltrate ↓ activity of myeloperoxidase ↓ MMP-3, MMP-13 (cartilage)
Milares et al. (2016)	ACLT	Rat	Exercise	λ(nm): 808 P (mW):50 Beam area (cm^2^): 0.028 T(s): 28/point, 2 points ED (J/cm^2^): 50 E(J):1.4 PD (W/cm^2^):1.7	3 days/week, 8 weeks	HA, IHC	↓ cartilage fibrillation and irregularities ↓ OARSI scores ↓ IL-1β, caspase-3, MMP-13 (cartilage)
Stancker et al. (2018)	Papain injection	Rat	ADSC	λ(nm): 808 P (mW):50 Beam area (cm^2^): 0.028 T(s):40/point, 4 points ED (J/cm^2^): 71.2 E(J):2 PD (W/cm^2^):1.78	1/day 7 days	Western blot, RT-PCR	↓ IL-1β, IL-6, TNF-α, MMPs(cartilage) ↑ TIMP-1, TIMP-2, IL-10, COL-2
Sanches et al. (2018)	ACLT	Rat	CS/GS	λ(nm): 808 P (mW):50 Beam area (cm^2^): 0.028 T(s):28/point, 2 points ED (J/cm^2^): 50 E(J):1.4 PD (W/cm^2^):1.7	3 days/week, 29 sessions	HA, IHC	↓ OARSI scores ↓ IL-1β(cartilage) ↑ IL-10, COL-2
Nambi (2021)	ACLT, MIA (review)	Rat	-	λ(nm): 780/805/808/830 P (mW):20/30/50/100 Beam area (cm^2^): 0.028/0.04 T(s): 20/28/40/47/60/80 ED (J/cm^2^): 10/50/71.4/142.4/144 /214.2 E(J): N/A PD (W/cm^2^):0.5/1.52/1.7/1.78/3.57	N/A	inflammatory cytokines analysis	↓ IL-1β, TNF-α, MMP-13 (cartilage) - IL-6
**Oxidative stress**							
Yamada et al. (2022)	MIA	Rat	STE	λ(nm): 904 P (mW):40 Beam area (cm^2^): 0.1309 T(s):27/point, 2 points ED (J/cm^2^): 18 E(J):N/A PD (mW/cm^2^):N/A	3 days/week 8 sessions	Nociception and edema evaluations, biochemical analyses ELISA	↓ nociception and oxidative stress ↓ NOx levels, IL-1β, TNF-α, IL-6 (knee structures:cruciate and collaterals ligaments, capsule, meniscus) ↑ the enzymatic activity of SOD
Martins et al. (2021)	MIA	Rat	-	λ(nm): 630 ± 20 P (mW):300 Beam area (cm^2^): 1 T(s):30 ED (J/cm^2^): 9 E(J):9 PD (W/cm^2^):0.3	3 days/week 8 weeks	HA, Determination of SOD activity, Determination of CAT activity, Determination of TBARS concentration, FRAP assay	↑ the preservation of chondrocytes ↑ SOD and CAT activities (red blood cell) ↓ oxidative damage ↓ TBARS
**Hyperalgesia**							
Balbinot et al. (2021)	MIA	Rat	-	λ(nm): 850 P (mW):100 Beam area (cm^2^): 0.07 T(s):40/point, 4 points ED (J/cm^2^): 57.14 E(J):4 PD (W/cm^2^):1.43	1/day 15 sessions	HA, behavioral test	↑ cartilage repair ↑ weight support ↓ pain ↓ spinal cord central sensitization ↓ widespread reactive astrogliosis
Wu et al. (2014)	MIA	Rat	-	λ(μm): 10.6 P (mW): N/A Beam area (cm^2^): N/A T(s): N/A ED (J/cm^2^): N/A E(J): N/A PD (mW/mm^2^):63.29	5min 1/day 8 days	Microarray analysis of cytokines, hindpaw eight-bearing	↓ pain ↓ Agrin, MMP-8 (spinal dorsal horn) ↑ TIMP-1
Li et al. (2020c)	MIA	Rat	-	λ(μm): 10.6 P (mW): N/A Beam area (cm^2^): N/A T (min):10 ED (J/cm^2^): 1,500 E(J):329.86 PD (mW/cm^2^):80	10min 1/day 7 days	behavior test, ELISA, HA, IHC	↓ nociceptive behaviors ↓ articular pathological lesions and cartilage destruction ↓ MMP-13 (cartilage), TNF-a, IL-1β, IL-6 (synovial membrane)
Li et al. (2020b)	MIA	Rat	-	λ(μm): 10.6 P (mW):80 Beam area (cm^2^): 0.0314 T (min):10 ED (J/cm^2^): 1,529 E(J):48.01 PD (mW/cm^2^):2,548	1/every 2 days 7 sessions	behavior test, Western blot, IF,ELISA	↓ nociceptive behaviors ↓ spinal microglial activation ↓ TNF-a, IL-1β, IL-6 (spinal cord)
Li et al. (2020a)	MIA	Rat	-	λ(μm): 10.6 P (mW):80 Beam area (cm^2^): 0.0314 T (min):10 ED (J/cm^2^): 1,529 E(J):48.01 PD (mW/cm^2^):2,548	1/day 7 days	behavior test, Western blot, IF, ELISA	↓ nociceptive behaviors ↓ p-NR1 (spinal cord) ↑ spinal A1R
Malta et al. (2022)	MIA	Mice	-	λ(nm): 830 P (mW):10 Beam area (cm^2^):0.1257 T (min):N/A ED (J/cm^2^): 8 E(J):1 PD (mW/cm^2^):N/A	21 days	Nociceptive test, gait assessment, joint temperature, and knee joint diameter, HA, ELISA	↓ nociceptive behaviors ↓ TNF-α(spinal cord) - gait, knee joint temperature, knee joint diameter
**Muscle atrophy**							
Assis et al. (2016)	ACLT	Rat	Exercise	λ(nm): 808 P (mW):50 Beam area (cm^2^): 0.028 T(s):28/point, 2 points ED (J/cm^2^): 50 E(J):1.4 PD (W/cm^2^):1.7	3 days/week, 8 weeks	HA, IHC	↓ cartilage fibrillation and irregularities ↓ OARSI scores ↓ IL-1β, caspase-3, MMP-13 (cartilage)
Assis et al. (2015)	ACLT	Rat	Exercise	λ(nm): 808 P (mW):50 Beam area (cm^2^): 0.028 T(s): 28/point, 2 points ED (J/cm^2^): 50 E(J):1.4 PD (mW/cm^2^):1.7	3 days/week, 8 weeks	HA, IHC, Muscle fiber density	↑ muscle cross-sectional area ↓ muscle fiber density (vastus medialis) ↓ MuRF-1, atrogin-1 (muscle cell)

MIA, monosodium iodoacetate; ACLT, anterior cruciate ligament transaction; ED, energy density; PD, power density; HA, histological analysis; IHC, immunohistological analysis; ELISA, Enzyme-Linked Immunosorbent Assay; IF, immunofluorescence; TUNEL, Terminal Deoxynucleotidyl Transferase dUTP, nick end labelling; FRAP, ferric reducing ability of plasma; LLLT:Low-Level Laser Therapy; ADSC, Adipose-Derived Stem Cell; NSAID, Non-Steroidal Anti-Inflammatory Drugs; COL, collagen; GAGs, Glycosaminoglycans; RT-PCR, Reverse Transcription Polymerase Chain Reaction; iNOS, i*nducible nitric oxide synthase*; TIMP, tissue inhibitor of metalloproteinases; TGF-β, Transforming Growth Factor-β; IL-1β/4/6/10, Interleukin-1β/4/6/10/13; MMP, metalloproteinases; TNF-α, Tumor Necrosis Factors-α; NO, nitric oxide; CS/GS, chondroitin sulfate and glucosamine sulfate; STE:S. tuberculata extract; SOD, superoxide dismutase; P-NR1, Phosphorylation of N-Methyl D-Aspartate Receptor 1; A1R, Adenosine A1 receptor; OARSI, osteoarthritis research society international; PGE2, Prostaglandin E2; CAT, catalase; TBARS, thiobarbituric acid reactive substances; Murf-1, Muscle RING-Finger Protein-1.

#### Cartilage degeneration

Cartilage degeneration is the main pathological feature of KOA, which caused by joint trauma, wear and inflammation (Pearle et al., 2005). Lin et al. found that 810 nm LLLT significantly improved the cartilage structure, reduced the disorganization and the expression of apoptosis related protein caspase-3 of chondrocytes in rabbit KOA (Lin et al., 2012). Oliveira et al. indicated that 830 nm LLLT modulated the chondrocyte proliferation and significantly decreased the expression of collagen (COL)-1 in rat KOA, but found no regulatory effect on inflammation (Oliveira et al., 2013). Moon et al. suggested that 4-week magnetic infrared laser (MIL) (850 nm LLLT with static magnetic field) had dose-dependent anti-inflammatory and cartilage protective effects on OA. They found that MIL inhibited cartilage degradation, increased the expression of cartilage glycosaminoglycans (GAGs), enhanced the proliferation of chondrocytes, improved the function of knee joint and alleviated edema (Moon et al., 2014). Oshima et al. demonstrated that LED with wavelengths of 630 nm (red) and 870 nm (infrared) has a chondroprotective effect, increasing the expression of COL-2 in cartilage and reduce the expression of TNF-α in cartilage and synovial tissue (Oshima et al., 2011). Trevisan et al. found that 850 nm LED can increase the expression of anabolic factor COL-2 and transforming growth factor (TGF)-β in cartilage (Trevisan et al., 2020). Milares et al. showed that LLLT had a positive effect on cartilage in KOA rats, mainly manifested by improved histology, decreased Osteoarthritis Research Society International (OARSI) score and density of chondrocytes, and higher cartilage thickness. A systematic review included three cellular studies and 30 animal studies, concluded that LLLT promotes extracellular matrix synthesis, reduces KOA pain and inflammation, and therefore has the potential to slow KOA progression and cartilage degeneration (Oliveira et al., 2021).

Although the aforementioned animal studies demonstrate a clear chondroprotective potential, a distinction must be made between these biological findings and clinical outcomes. In clinical settings, PBM is primarily recognized for its symptom-modifying effects, with strong evidence supporting improvements in VAS and WOMAC scores via anti-inflammatory pathways. In contrast, evidence for cartilage regeneration in human subjects is less definitive and remains secondary. For instance, Vassão et al. found that while PBM combined with exercise significantly increased the anti-inflammatory cytokine IL-10, it failed to produce significant changes in CTX-II (C-telopeptide of type II collagen), a specific biomarker for cartilage degradation (Vassão et al., 2021a). This discrepancy highlights that while PBM effectively modulates the inflammatory microenvironment, definitive clinical evidence of joint space widening or structural reversal is currently limited. Therefore, current evidence supports PBM as a tool to potentially slow progression by improving the joint environment, rather than a proven regenerative cure.

#### Inflammation

KOA is a chronic low-level inflammatory disease, and the increased release of pro-inflammatory and catabolic cytokines accelerate the degradation of joint cartilage and exacerbate the inflammatory response within the joint (Hunter and Bierma-Zeinstra, 2019). Wang et al. found that 6 weeks of 830 nm LLLT treatment can reduce the expression of inflammatory and catabolic factors such as IL-1β, inducible nitric oxide synthase (iNOS), and MMP-3, and increase the expression of anabolic factors such as tissue inhibitor of metalloproteinase (TIMP)-1, and 8 weeks of LLLT treatment can delay the loss of COL-2, aggrecan, and the anabolic factor transforming growth factor (TGF)-β (Wang et al., 2014). Tim et al. suggested that 808 nm LLLT increased the expression of anabolic factors such as IL-4, IL-10, COL-2, aggrecan and TGF-β in the cartilage tissue of KOA rats, and decreased the expression of inflammatory factor IL-1β, thereby promoting cartilage repair (Tim et al., 2022). Yamada et al. found that 904 nm LLLT can reduce the activity of neutrophils, decrease the levels of NO and cytokines (including TNF-α, IL-1β and IL-6), and reduce pain in KOA rats (Yamada et al., 2020). The study by da Rosa et al. induced KOA model of rats by injecting papain, and then intervened with LLLT at wavelengths of 660 and 808 nm, respectively. The results showed that LLLT at 808 nm could significantly stimulate angiogenesis, reduce inflammatory exudation and cartilage fibrosis, thereby produce a repair effect on KOA cartilage (da Rosa et al., 2012). Tomazoni et al. found that compared with exercise and topical sodium diclofenac, LLLT alone significantly reduced the morphological changes in cartilage tissue caused by OA, and decreased the expression of MMP-3, MMP-1, and pro-inflammatory cytokines IL-1β, IL-6, TNF-α, and PGE2 (Tomazoni et al., 2016; Tomazoni et al., 2017). Milares et al. found that LLLT significantly decreased the expression of IL-1β, and LLLT combined with exercise training significantly decreased the expression of MMP-13 (Milares et al., 2016). Stancker et al. found that both LLLT and adipose-derived stem cells (ADSCs) intra-articular injection could reduce the levels of pro-inflammatory cytokines (IL-1β, IL-6 and TNF-α) and MMPs, and increased the expression of TIMP-1, TIMP-2, IL-10 and COL-2 in KOA cartilage, and the combination of LLLT and ADSCs is more effective (Stancker et al., 2018). Sanches et al. demonstrated that chondroitin sulfate and glucosamine sulfate (CS/GS) combined with LLLT can significantly reduce the expression of IL-1β and increase the expression of IL-10 and COL-2 in cartilage of KOA rats (Sanches et al., 2018). A systematic review and meta-analysis involving 8 animal studies showed that LLLT could significantly reduce the levels of IL-1β, TNF-α and MMP-13 in knee joint of KOA rats, and regulate the proliferation of inflammatory cells, but had no significant effect on the levels of IL-6 (Nambi, 2021).

While preclinical studies clearly demonstrate that PBM downregulates pro-inflammatory cytokines, linking these molecular changes to clinical outcomes requires critical interpretation. The strong analgesic effects observed in clinical trials (VAS score reduction) are likely a direct downstream result of this anti-inflammatory modulation. However, the complex heterogeneous nature of human OA pathology means that the robust cartilage repair seen in controlled animal models may not translate linearly to human patients without optimized dosimetry. Therefore, current clinical evidence should be viewed as providing strong support for inflammatory modulation, while the evidence for complete structural restoration remains emerging.

#### Oxidative stress

Oxidative stress plays an important role in KOA, and studies have found that oxidative stress not only promotes the aging and damage of chondrocytes, but also promotes the development of synovitis (Liu et al., 2022). Yamada et al. found that LLLT reduced the levels of oxidative stress markers in serum and spinal cord, and enhanced the antioxidant capacity of serum and brainstem of KOA rats (Yamada et al., 2022). Martins et al. investigated the LED on oxidative stress and histological aspects of KOA rats. Their results showed that 630 nm LED can increase cartilage thickness and chondrocyte number in KOA rats, activate antioxidant enzymes superoxide dismutase (SOD) and catalase (CAT), and reduce the concentration of thiobarbituric acid reactive substances (TBARS), a biomarker of oxidative stress damage. Therefore, LED can promote cartilage repair and reduce oxidative damage caused by lipid peroxidation (Martins et al., 2021).

While preclinical data strongly suggest that PBM mitigates oxidative damage at the cellular level, the direct clinical evidence linking antioxidant enzyme activation to disease modification in human KOA remains speculative. In animal models, the controlled environment allows for precise measurement of reactive oxygen species (ROS) and enzymatic activity. However, in clinical practice, it is challenging to isolate the specific contribution of “reduced oxidative stress” to the overall therapeutic outcome, as it is often overshadowed by the more immediate anti-inflammatory effects. Therefore, while PBM-induced antioxidant activity is a biologically plausible mechanism for slowing disease progression, it currently lacks the high-level clinical validation needed to be classified as a primary “confirmed” mechanism in humans.

#### Hyperalgesia

Peripheral receptors around the knee joint convert noxious stimuli into neural impulses that converge on the dorsal horn of the spinal cord, where cells release algogenic and inflammatory factors. These signals are relayed through the brainstem and hypothalamus to the limbic system and cerebral cortex, resulting in hyperalgesia (Güngör Demir et al., 2021). Balbinot et al. suggested that 850 nm LLLT could promote the recovery of cartilage repair and weight support, and reduce spinal cord central sensitization and improve pain, these effects were associated with the reduction of widespread reactive astrogliosis in the spinal cord dorsal horn (Balbinot et al., 2021). Wu et al. found that 10.6 μm CO_2_ laser irradiation on acupoint Dubi significantly reduced pain, increased TIMP-1 expression and decreased Agrin and MMP-8 expression in the ipsilateral spinal dorsal horn of KOA rat (Wu et al., 2014). Li et al. showed that early intervention of laser irradiation at ST35 could significantly reduce the levels of cartilage MMP-13 and synovial TNF-α, IL-1β and IL-6, and inhibit the activation of microglia and the upregulation of pro-inflammatory cytokines TNF-α, IL-1β and IL-6 at the spinal level. Thereby inhibiting neuroinflammation and central sensitization. In addition, laser irradiation at ST35 can upregulate spinal adenosine A1 receptor to inhibit nociception and phosphorylation of N-methyl D-aspartate receptor 1 (p-NR1), thereby relieving pain. These results indicate that laser has analgesic, anti-inflammatory and cartilage protective effects (Li et al., 2020c; 2020b; 2020a). The study by Malta et al. suggested that spinal microglia and astrocytes in OA would release TNF-α to induce nociception and neuroglial activation, while LLLT could reduce nociception and TNF-α release, and there was no significant difference compared to ultrasound therapy (Malta et al., 2022).

The evidence supporting PBM as a tool for modulating hyperalgesia is robust and clinically significant. There is a clear, evidence-based link between the inhibition of spinal neuroinflammation observed in preclinical models and the long-term analgesic benefits reported in human RCTs. Unlike cartilage regeneration, which is difficult to quantify clinically, the reduction in hyperalgesia can be directly measured through Pressure Pain Thresholds (PPT) and VAS scores. This suggests that PBM’s efficacy extends beyond local joint treatment; it acts on the nervous system’s pain-processing pathways. The strong correlation between laboratory findings of reduced central sensitization and clinical reports of sustained pain relief provides solid evidence for PBM’s role as a potent neuromodulatory intervention for KOA pain management.

#### Muscle atrophy

Quadriceps muscle mass and strength determine lower extremity functions, and quadriceps muscle atrophy has been demonstrated in patients with KOA. Additionally, greater quadriceps strength protects against cartilage degeneration (Kim et al., 2023). Assis et al. revealed that LLLT exhibited significant potential in reducing fibrosis and irregularity of the articular cartilage surface in rats with KOA. Additionally, they found that LLLT effectively decreased the expression of IL-1β, caspase-3 and MMP-13 in cartilage, as well as muscle protein degradation markers atrogin-1 and muscle-specific ring-finger protein 1 (MuRF-1) in vastus medialis (VM), indicating the anti-inflammatory properties, ability to prevent muscle atrophy and cartilage degeneration. Furthermore, LLLT combined with exercise demonstrated a more pronounced effect on anti-inflammation and preventing cartilage degeneration, nevertheless, no additive effect is observed regarding preventing muscle atrophy (Assis et al., 2015; 2016).

While PBM shows potential in preventing muscle atrophy in preclinical settings, clinical evidence suggests that PBM is most effective when used as an adjunct to exercise, rather than a standalone treatment for muscle strengthening. The “prevention of atrophy” is likely secondary to pain reduction, which allows patients to engage in more vigorous physical therapy, thereby improving clinical outcomes through a synergistic “biophysical-mechanical” pathway.

The above studies explained the cartilage protection, anti-inflammatory, muscle atrophy prevention and analgesic effects of PBM on KOA from the aspects of cartilage matrix metabolism, inflammatory factor levels, spinal cord central sensitization, oxidative stress injury and muscle protein degradation. Due to the complex pathogenesis of KOA, these studies were phenotypic studies and did not explore the molecular mechanism of PBM for KOA from signal transduction. In addition, the mechanism of PBM combined with other therapies, such as exercise, still needs to be further explored.

## Conclusion

It is imperative to distinguish between symptom modulation and disease modification in the context of PBM for KOA. There is strong and consistent evidence supporting PBM as an effective modality for immediate and short-term symptomatic relief, particularly in mitigating pain and inflammation. However, evidence for cartilage regeneration in human subjects remains emerging and partly speculative, as it is primarily extrapolated from preclinical histological findings. While PBM creates a favorable environment for chondroprotection by downregulating catabolic markers (e.g., MMP-13), definitive clinical evidence of joint space widening or structural reversal is currently lacking.

In summary, while numerous studies have investigated the therapeutic potential of PBM for KOA, the results remain heterogeneous. Some trials demonstrate significant improvements in pain and disability, whereas others report negligible effects. These discrepancies are largely attributable to the absence of standardized treatment protocols. Variations in key parameters-such as wavelength, energy density, and treatment duration-can lead to divergent clinical outcomes. Furthermore, as PBM technologies evolve, the distinct roles of LLLT, HILT, and LED must be further elucidated. In particular, despite the promise of LED as a low-cost, wearable, and accessible intervention, its clinical evidence base remains relatively limited.

Consequently, future research should prioritize large-scale, multi-center clinical trials with long-term follow-up to determine optimal, standardized parameters for KOA. Additionally, while current *in vitro* and *in vivo* studies have identified several phenotypic changes, including improvements in cartilage matrix metabolism and reductions in oxidative stress and central sensitization, the underlying signal transduction pathways remain partially understood. Transitioning from phenotypic observation to deep mechanistic exploration of molecular signaling will be essential to refine PBM devices and provide more robust, evidence-based justification for its clinical application in KOA management.

## Glossary

ATP: Adenosine triphosphate
ADSCs: Adipose-derived stem cells
CTX-II: Collagen type-II C telopeptide
COX-2: Cyclooxygenase-2
CPT: Conservative physical therapy
CS: Chondroitin sulfate
COL: Collagen
CAT: Catalase
ESWT: Extracorporeal shockwave therapy
FDA: Food and Drug Administration
GS: Glucosamine sulfate
GAGs: Cartilage glycosaminoglycans
HILT: High-intensity laser therapy
IL-1β: Interleukin 1β
IFC: Interferential current
iNOS: Inducible nitric oxide synthase
KOA: Knee osteoarthritis
KOOS: Knee injury and Osteoarthritis Outcome Score
LED: Light-emitting diodes
LLLT: Low-level laser therapy
MAPK: Mitogen-activated protein kinases
MMP: matrix metalloproteinase
MIRE: Monochromatic infrared energy
MIL: Magnetic infrared laser
MuRF-1: Muscle-specific ring-finger protein 1
NO: Nitric oxide
NF-κB: Nuclear factor-kappa B
NES: Neuromuscular electrical stimulation
OARSI: Osteoarthritis Research Society International
PBM: Photobiomodulation
PEMF: Pulsed electromagnetic field
PEDro: Physiotherapy Evidence Database
PRKD2: Protein kinase D2
PGE2: Prostaglandin E2
p-NR1: phosphorylation of N-methyl D-aspartate receptor 1
RCT: Randomized controlled trial
ROS: Reactive oxygen species
ROM: Range of motion
SOD: Superoxide dismutase
ΤΝF-α: Tumor necrosis factor-α
TENS: Transcutaneous electric nerve stimulation
TGF: Transforming growth factor
TIMP: Tissue inhibitor of metalloproteinase
TBARS: Thiobarbituric acid reactive substances
VM: Vastus medialis
WBV: Whole-body vibration
WOMAC: Western Ontario and McMaster Universities osteoarthritis index
VM: Vastus medialis

## References

[B1] Ahmad M. A. Hamid A. Yusof A. (2022). Effects of low-level and high-intensity laser therapy as adjunctive to rehabilitation exercise on pain, stiffness and function in knee osteoarthritis: a systematic review and meta-analysis. Physiotherapy 114, 85–95. 10.1016/j.physio.2021.03.011 34654554

[B2] Akaltun M. S. Altindag O. Turan N. Gursoy S. Gur A. (2021). Efficacy of high intensity laser therapy in knee osteoarthritis: a double-blind controlled randomized study. Clin. Rheumatol. 40, 1989–1995. 10.1007/s10067-020-05469-7 33074393

[B3] Al Rashoud A. S. Abboud R. J. Wang W. Wigderowitz C. (2014). Efficacy of low-level laser therapy applied at acupuncture points in knee osteoarthritis: a randomised double-blind comparative trial. Physiotherapy 100, 242–248. 10.1016/j.physio.2013.09.007 24418801

[B4] Alayat M. S. M. Aly T. H. A. Elsayed A. E. M. Fadil A. S. M. (2017). Efficacy of pulsed Nd:YAG laser in the treatment of patients with knee osteoarthritis: a randomized controlled trial. Lasers Med. Sci. 32, 503–511. 10.1007/s10103-017-2141-x 28078503

[B5] Alfredo P. P. Bjordal J. M. Dreyer S. H. Meneses S. R. Zaguetti G. Ovanessian V. (2012). Efficacy of low level laser therapy associated with exercises in knee osteoarthritis: a randomized double-blind study. Clin. Rehabil. 26, 523–533. 10.1177/0269215511425962 22169831

[B6] Alfredo P. P. Bjordal J. M. Junior W. S. Lopes-Martins Rá B. Stausholm M. B. Casarotto R. A. (2018). Long-term results of a randomized, controlled, double-blind study of low-level laser therapy before exercises in knee osteoarthritis: laser and exercises in knee osteoarthritis. Clin. Rehabil. 32, 173–178. 10.1177/0269215517723162 28776408

[B7] Alfredo P. P. Bjordal J. M. Lopes-Martins Rá B. Johnson M. I. Junior W. S. Marques A. P. (2022). Efficacy of prolonged application of low-level laser therapy combined with exercise in knee osteoarthritis: a randomized controlled double-blind study. Clin. Rehabil. 36, 1281–1291. 10.1177/02692155221111922 35918813

[B8] Alghadir A. Omar M. T. Al-Askar A. B. Al-Muteri N. K. (2014). Effect of low-level laser therapy in patients with chronic knee osteoarthritis: a single-blinded randomized clinical study. Lasers Med. Sci. 29, 749–755. 10.1007/s10103-013-1393-3 23912778

[B9] Alqualo-Costa R. Rampazo É P. Thome G. R. Perracini M. R. Liebano R. E. (2021). Interferential current and photobiomodulation in knee osteoarthritis: a randomized, placebo-controlled, double-blind clinical trial. Clin. Rehabil. 35, 1413–1427. 10.1177/02692155211012004 33896234

[B10] Ammar T. A. (2014). Monochromatic infrared photo energy versus low level laser therapy in patients with knee osteoarthritis. J. Lasers Med. Sci. 5, 176–182. 25653818 PMC4281991

[B11] Ammendolia A. Marotta N. Marinaro C. Demeco A. Mondardini P. Costantino C. (2021). The synergic use of the high power Laser Therapy and Glucosamine sulfate in knee osteoarthritis: a randomized controlled trial. Acta Biomed. 92, e2021237. 10.23750/abm.v92i3.10952 34212917 PMC8343723

[B12] Angelova A. Ilieva E. M. (2016). Effectiveness of high intensity laser therapy for reduction of pain in knee osteoarthritis. Pain Res. Manag. 2016, 9163618. 10.1155/2016/9163618 28096711 PMC5206453

[B13] Assis L. Almeida T. Milares L. P. dos Passos N. Araújo B. Bublitz C. (2015). Musculoskeletal atrophy in an experimental model of knee osteoarthritis: the effects of exercise training and low-level laser therapy. Am. J. Phys. Med. Rehabil. 94, 609–616. 10.1097/phm.0000000000000219 25299541

[B14] Assis L. Milares L. P. Almeida T. Tim C. Magri A. Fernandes K. R. (2016). Aerobic exercise training and low-level laser therapy modulate inflammatory response and degenerative process in an experimental model of knee osteoarthritis in rats. Osteoarthr. Cartil. 24, 169–177. 10.1016/j.joca.2015.07.020 26254236

[B15] Balbinot G. Schuch C. P. Nascimento P. S. D. Lanferdini F. J. Casanova M. Baroni B. M. (2021). Photobiomodulation therapy partially restores cartilage integrity and reduces chronic pain behavior in a rat model of osteoarthritis: involvement of spinal glial modulation. Cartilage 13, 1309s–1321s. 10.1177/1947603519876338 31569995 PMC8804719

[B16] Barolet D. (2008). Light-emitting diodes (LEDs) in dermatology. Semin. Cutan. Med. Surg. 27, 227–238. 10.1016/j.sder.2008.08.003 19150294

[B17] Bian J. Liebert A. Bicknell B. Chen X.-M. Huang C. Pollock C. A. (2022). Therapeutic potential of photobiomodulation for chronic kidney disease. Int. J. Mol. Sci. 23, 8043. 10.3390/ijms23148043 35887386 PMC9320354

[B18] Brosseau L. Robinson V. Wells G. Debie R. Gam A. Harman K. (2005). Low level laser therapy (classes I, II and III) for treating rheumatoid arthritis. Cochrane Database Syst. Rev. 2005, CD002049. 10.1002/14651858.CD002049.pub2 16235295 PMC8406947

[B19] Bülow P. M. Jensen H. Danneskiold-Samsøe B. (1994). Low power ga-al-as laser treatment of painful osteoarthritis of the knee. A double-blind placebo-controlled study. Scand. J. Rehabil. Med. 26, 155–159. 7801065

[B20] Bunch J. (2023). Photobiomodulation (therapeutic lasers): an update and review of current literature. Vet. Clin. North Am. Small Anim. Pract. 53, 783–799. 10.1016/j.cvsm.2023.02.010 36964028

[B21] Burke T. J. (2003). 5 Questions-and answers-about MIRE treatment. Adv. Skin. Wound Care 16, 369–371. 10.1097/00129334-200312000-00016 14688646

[B22] Cao P. Li Y. Tang Y. Ding C. Hunter D. J. (2020). Pharmacotherapy for knee osteoarthritis: current and emerging therapies. Expert Opin. Pharmacother. 21, 797–809. 10.1080/14656566.2020.1732924 32100600

[B23] Charlier E. Deroyer C. Ciregia F. Malaise O. Neuville S. Plener Z. (2019). Chondrocyte dedifferentiation and osteoarthritis (OA). Biochem. Pharmacol. 165, 49–65. 10.1016/j.bcp.2019.02.036 30853397

[B24] Chen Z. Ma C. Xu L. Wu Z. He Y. Xu K. (2019). Laser acupuncture for patients with knee osteoarthritis: a systematic review and meta-analysis of randomized placebo-controlled trials. Evid. Based Complement. Altern. Med. 2019, 6703828. 10.1155/2019/6703828 31781275 PMC6874873

[B25] Chung H. Dai T. Sharma S. K. Huang Y.-Y. Carroll J. D. Hamblin M. R. (2012). The nuts and bolts of low-level laser (light) therapy. Ann. Biomed. Eng. 40, 516–533. 10.1007/s10439-011-0454-7 22045511 PMC3288797

[B26] Cui A. Li H. Wang D. Zhong J. Chen Y. Lu H. (2020). Global, regional prevalence, incidence and risk factors of knee osteoarthritis in population-based studies. EClinicalMedicine 29–30, 100587. 10.1016/j.eclinm.2020.100587 34505846 PMC7704420

[B27] da Rosa A. S. dos Santos A. F. da Silva M. M. Facco G. G. Perreira D. M. Alves A. C. (2012). Effects of low-level laser therapy at wavelengths of 660 and 808 nm in experimental model of osteoarthritis. Photochem Photobiol. 88, 161–166. 10.1111/j.1751-1097.2011.01032.x 22053992

[B28] de Freitas L. F. Hamblin M. R. (2016). Proposed mechanisms of photobiomodulation or low-level light therapy. IEEE Journal Selected Topics Quantum Electronics A Publication IEEE Lasers Electro-optics Soc. 22, 7000417. 10.1109/JSTQE.2016.2561201 28070154 PMC5215870

[B29] de Matos Brunelli Braghin R. Libardi E. C. Junqueira C. Rodrigues N. C. Nogueira-Barbosa M. H. Renno A. C. M. (2019). The effect of low-level laser therapy and physical exercise on pain, stiffness, function, and spatiotemporal gait variables in subjects with bilateral knee osteoarthritis: a blind randomized clinical trial. Disabil. Rehabil. 41, 3165–3172. 10.1080/09638288.2018.1493160 30324827

[B30] de Oliveira Melo M. Pompeo K. D. Baroni B. M. Vaz M. A. (2016). Effects of neuromuscular electrical stimulation and low-level laser therapy on neuromuscular parameters and health status in elderly women with knee osteoarthritis: a randomized trial. J. Rehabil. Med. 48, 293–299. 10.2340/16501977-2062 26871692

[B31] de Paula Gomes C. A. F. Leal-Junior E. C. P. Dibai-Filho A. V. de Oliveira A. R. Bley A. S. Biasotto-Gonzalez D. A. (2018). Incorporation of photobiomodulation therapy into a therapeutic exercise program for knee osteoarthritis: a placebo-controlled, randomized, clinical trial. Lasers Surg. Med. 50, 819–828. 10.1002/lsm.22939 29733117

[B32] Dias S. B. F. Fonseca M. V. A. Dos Santos N. C. C. Mathias I. F. Martinho F. C. Junior M. S. (2015). Effect of GaAIAs low-level laser therapy on the healing of human palate mucosa after connective tissue graft harvesting: randomized clinical trial. Lasers Med. Sci. 30, 1695–1702. 10.1007/s10103-014-1685-2 25373688

[B33] Dompe C. Moncrieff L. Matys J. Grzech-Leśniak K. Kocherova I. Bryja A. (2020). Photobiomodulation—Underlying mechanism and clinical applications. JCM 9, 1724. 10.3390/jcm9061724 32503238 PMC7356229

[B34] Elboim-Gabyzon M. Nahhas F. (2023). Laser therapy versus pulsed electromagnetic field therapy as treatment modalities for early knee osteoarthritis: a randomized controlled trial. BMC Geriatr. 23, 144. 10.1186/s12877-022-03568-5 36922781 PMC10018856

[B35] Elvir-Lazo O. L. Yumul R. White P. F. (2020). Cold laser therapy for acute and chronic pain management. Top. Pain Manag. 36, 1–10. 10.1097/01.TPM.0000696768.75244.e0

[B36] Fakhari S. Pishghahi A. Pourfathi H. Farzin H. Bilehjani E. (2021). A comparison between low-level laser therapy and intra-articular ozone injection in knee osteoarthritis treatment: a randomized clinical trial. J. Lasers Med. Sci. 12, e44. 10.34172/jlms.2021.44 34733767 PMC8558735

[B37] Fl H. J C. C L. M C. A L. A M. (2009). Laser and other light therapies for the treatment of acne vulgaris: systematic review. Br. Journal Dermatology 160. 10.1111/j.1365-2133.2009.09047.x 19239470

[B38] Foo A. S. C. Soong T. W. Yeo T. T. Lim K.-L. (2020). Mitochondrial dysfunction and parkinson’s disease-near-infrared photobiomodulation as a potential therapeutic strategy. Front. Aging Neurosci. 12, 89. 10.3389/fnagi.2020.00089 32308618 PMC7145956

[B39] Fukuda V. O. Fukuda T. Y. Guimarães M. Shiwa S. de Lima Bdel C. Martins R. (2011). Short-term efficacy of low-level laser therapy in patients with knee osteoarthritis: a randomized placebo-controlled, double-blind clinical trial. Rev. Bras. Ortop. 46, 526–533. 10.1016/s2255-4971(15)30407-9 27027049 PMC4799277

[B40] Gendron D. J. Hamblin M. R. (2019). Applications of photobiomodulation therapy to musculoskeletal disorders and osteoarthritis with particular relevance to Canada. Photobiomodul Photomed. Laser Surg. 37, 408–420. 10.1089/photob.2018.4597 31265376 PMC6648198

[B41] Giorgino R. Albano D. Fusco S. Peretti G. M. Mangiavini L. Messina C. (2023). Knee osteoarthritis: epidemiology, pathogenesis, and mesenchymal stem cells: what else is new? An update. Int. J. Mol. Sci. 24, 6405. 10.3390/ijms24076405 37047377 PMC10094836

[B42] Glass G. E. (2021a). Photobiomodulation: a review of the molecular evidence for low level light therapy. J. Plast. Reconstr. Aesthet. Surg. 74, 1050–1060. 10.1016/j.bjps.2020.12.059 33436333

[B43] Glass G. E. (2021b). Photobiomodulation: the clinical applications of low-level light therapy. Aesthetic Surg. J. 41, 723–738. 10.1093/asj/sjab025 33471046

[B44] Glazov G. Yelland M. Emery J. (2016). Low-level laser therapy for chronic non-specific low back pain: a meta-analysis of randomised controlled trials. Acupunct. Med. 34, 328–341. 10.1136/acupmed-2015-011036 27207675 PMC5099186

[B45] Gopal S. Kamal W. George J. Manssor E. (2017). Radiological and biochemical effects (CTX-II, MMP-3, 8, and 13) of low-level laser therapy (LLLT) in chronic osteoarthritis in Al-Kharj, Saudi Arabia. Lasers Med. Sci. 32, 297–303. 10.1007/s10103-016-2114-5 27913970

[B46] Güngör Demir U. Demir A. N. Toraman N. F. (2021). Neuropathic pain in knee osteoarthritis. Adv. Rheumatol. 61, 67. 10.1186/s42358-021-00225-0 34743761

[B47] Gur A. Cosut A. Sarac A. J. Cevik R. Nas K. Uyar A. (2003). Efficacy of different therapy regimes of low-power laser in painful osteoarthritis of the knee: a double-blind and randomized-controlled trial. Lasers Surg. Med. 33, 330–338. 10.1002/lsm.10236 14677160

[B48] Gworys K. Gasztych J. Puzder A. Gworys P. Kujawa J. (2012). Influence of various laser therapy methods on knee joint pain and function in patients with knee osteoarthritis. Ortop. Traumatol. Rehabil. 14, 269–277. 10.5604/15093492.1002257 22764339

[B49] Hamblin M. R. (2017). Mechanisms and applications of the anti-inflammatory effects of photobiomodulation. AIMS Biophysics 4, 337–361. 10.3934/biophy.2017.3.337 28748217 PMC5523874

[B50] Hawker G. A. (2019). Osteoarthritis is a serious disease. Clin. Exp. Rheumatol. 37 (Suppl. 120), 3–6. 31621562

[B51] Hegedus B. Viharos L. Gervain M. Gálfi M. (2009). The effect of low-level laser in knee osteoarthritis: a double-blind, randomized, placebo-controlled trial. Photomed. Laser Surg. 27, 577–584. 10.1089/pho.2008.2297 19530911 PMC2957068

[B52] Heiskanen V. Hamblin M. R. (2018). Photobiomodulation: lasers vs. light emitting diodes? Photochem Photobiol. Sci. 17, 1003–1017. 10.1039/c8pp90049c 30044464 PMC6091542

[B53] Helianthi D. R. Simadibrata C. Srilestari A. Wahyudi E. R. Hidayat R. (2016). Pain reduction after laser acupuncture treatment in geriatric patients with knee osteoarthritis: a randomized controlled trial. Acta Med. Indones. 48, 114–121. 27550880

[B54] Hennessy M. Hamblin M. R. (2017). Photobiomodulation and the brain: a new paradigm. J. Opt. 19, 013003. 10.1088/2040-8986/19/1/013003 28580093 PMC5448311

[B55] Hsieh R. L. Lo M. T. Lee W. C. Liao W. C. (2012). Therapeutic effects of short-term monochromatic infrared energy therapy on patients with knee osteoarthritis: a double-blind, randomized, placebo-controlled study. J. Orthop. Sports Phys. Ther. 42, 947–956. 10.2519/jospt.2012.3881 22960644

[B56] Huang Z. Chen J. Ma J. Shen B. Pei F. Kraus V. B. (2015). Effectiveness of low-level laser therapy in patients with knee osteoarthritis: a systematic review and meta-analysis. Osteoarthr. Cartil. 23, 1437–1444. 10.1016/j.joca.2015.04.005 25914044 PMC4814167

[B57] Hunter D. J. Bierma-Zeinstra S. (2019). Osteoarthr. Lancet London, Engl. 393, 1745–1759. 10.1016/S0140-6736(19)30417-9 31034380

[B58] Jankaew A. You Y. L. Yang T. H. Chang Y. W. Lin C. F. (2023). The effects of low-level laser therapy on muscle strength and functional outcomes in individuals with knee osteoarthritis: a double-blinded randomized controlled trial. Sci. Rep. 13, 165. 10.1038/s41598-022-26553-9 36599881 PMC9812996

[B59] Karu T. (1999). Primary and secondary mechanisms of action of visible to near-IR radiation on cells. J. Photochem Photobiol. B 49, 1–17. 10.1016/S1011-1344(98)00219-X 10365442

[B60] Kauark-Fontes E. Migliorati C. A. Epstein J. B. Bensadoun R.-J. Gueiros L. A. M. Carroll J. (2023). Twenty-year analysis of photobiomodulation clinical studies for oral mucositis: a scoping review. Oral Surg. Oral Med. Oral Pathol. Oral Radiol. 135, 626–641. 10.1016/j.oooo.2022.12.010 36870898

[B61] Kh L. D L. Yt C. Sy C. (2019). Comparative effectiveness of low-level laser therapy for adult androgenic alopecia: a system review and meta-analysis of randomized controlled trials. Lasers Medical Science 34. 10.1007/s10103-019-02723-6 30706177

[B62] Kheshie A. R. Alayat M. S. Ali M. M. (2014). High-intensity *versus* low-level laser therapy in the treatment of patients with knee osteoarthritis: a randomized controlled trial. Lasers Med. Sci. 29, 1371–1376. 10.1007/s10103-014-1529-0 24487957

[B63] Kholvadia A. Constantinou D. Gradidge P. J. (2019). Exploring the efficacy of low-level laser therapy and exercise for knee osteoarthritis. S Afr. J. Sports Med. 31, v31i1a6058. 10.17159/2078-516X/2019/v31i1a6058 36817986 PMC9924591

[B64] Kim G. J. Choi J. Lee S. Jeon C. Lee K. (2016). The effects of high intensity laser therapy on pain and function in patients with knee osteoarthritis. J. Phys. Ther. Sci. 28, 3197–3199. 10.1589/jpts.28.3197 27942148 PMC5140828

[B65] Kim J.-R. Pham T. H. N. Kim W.-U. Kim H. A. (2023). A causative role for periarticular skeletal muscle weakness in the progression of joint damage and pain in OA. Sci. Rep. 13, 21349. 10.1038/s41598-023-46599-7 38049482 PMC10696078

[B66] Letizia Mauro G. Scaturro D. Gimigliano F. Paoletta M. Liguori S. Toro G. (2021). Physical agent modalities in early osteoarthritis: a scoping review. Med. Kaunas. 57, 1165. 10.3390/medicina57111165 34833383 PMC8619194

[B67] Li Y. Wu F. Lao L. X. Shen X. Y. (2020a). Laser irradiation activates spinal adenosine A1 receptor to alleviate osteoarthritis pain in monosodium iodoacetate injected rats. J. Integr. Neurosci. 19, 295–302. 10.31083/j.jin.2020.02.33 32706193

[B68] Li Y. Wu F. Wei J. Lao L. Shen X. (2020b). Laser moxibustion alleviates knee osteoarthritis pain by inhibiting spinal microglial activation-mediated neuroinflammation in rats. Photobiomodul Photomed. Laser Surg. 38, 237–243. 10.1089/photob.2019.4744 31976816

[B69] Li Y. Wu F. Wei J. Lao L. Shen X. (2020c). The effects of laser moxibustion on knee Osteoarthritis pain in rats. Photobiomodul Photomed. Laser Surg. 38, 43–50. 10.1089/photob.2019.4716 31549920 PMC6978776

[B70] Liao F. Y. Lin C. L. Lo S. F. Chang C. C. Liao W. Y. Chou L. W. (2020). Efficacy of acupoints dual-frequency low-level laser therapy on knee osteoarthritis. Evid. Based Complement. Altern. Med. 2020, 6979105. 10.1155/2020/6979105 33029170 PMC7532399

[B71] Lin H. D. He C. Q. Luo Q. L. Zhang J. L. Zeng D. X. (2012). The effect of low-level laser to apoptosis of chondrocyte and caspases expression, including caspase-8 and caspase-3 in rabbit surgery-induced model of knee osteoarthritis. Rheumatol. Int. 32, 759–766. 10.1007/s00296-010-1629-5 21188382

[B72] Lin L. Cheng K. Tan M. T. Zhao L. Huang Z. Yao C. (2020). Comparison of the effects of 10.6-mum infrared laser and traditional moxibustion in the treatment of knee osteoarthritis. Lasers Med. Sci. 35, 823–832. 10.1007/s10103-019-02863-9 31446581 PMC7260151

[B73] Liu L. Luo P. Yang M. Wang J. Hou W. Xu P. (2022). The role of oxidative stress in the development of knee osteoarthritis: a comprehensive research review. Front. Mol. Biosci. 9, 1001212. 10.3389/fmolb.2022.1001212 36203877 PMC9532006

[B74] Maher C. G. Sherrington C. Herbert R. D. Moseley A. M. Elkins M. (2003). Reliability of the PEDro scale for rating quality of randomized controlled trials. Phys. Ther. 83, 713–721. 12882612

[B75] Malik S. Sharma S. Dutta N. Khurana D. Sharma R. K. Sharma S. (2023). Effect of low-level laser therapy plus exercise therapy on pain, range of motion, muscle strength, and function in knee osteoarthritis - a systematic review and meta-analysis. Somatosens. Mot. Res. 40, 8–24. 10.1080/08990220.2022.2157387 36576096

[B76] Malta I. Moraes T. Elisei L. Novaes R. Galdino G. (2022). Investigation of the effects of therapeutic ultrasound or photobiomodulation and the role of spinal glial cells in osteoarthritis-induced nociception in mice. Lasers Med. Sci. 37, 1687–1698. 10.1007/s10103-021-03418-7 34542770

[B77] Martins L. P. O. Santos F. F. D. Costa T. E. D. Lacerda A. C. R. Santos J. M. D. Costa K. B. (2021). Photobiomodulation therapy (Light-Emitting diode 630 nm) favored the oxidative stress and the preservation of articular cartilage in an induced knee Osteoarthritis model. Photobiomodul Photomed. Laser Surg. 39, 272–279. 10.1089/photob.2020.4926 33497593

[B78] Melo Mde O. Pompeo K. D. Brodt G. A. Baroni B. M. da Silva Junior D. P. Vaz M. A. (2015). Effects of neuromuscular electrical stimulation and low-level laser therapy on the muscle architecture and functional capacity in elderly patients with knee osteoarthritis: a randomized controlled trial. Clin. Rehabil. 29, 570–580. 10.1177/0269215514552082 25261425

[B79] Mester E. Szende B. Gärtner P. (1968). The effect of laser beams on the growth of hair in mice. Radiobiol. Radiother. Berl. 9, 621–626. 5732466

[B80] Mester E. Spiry T. Szende B. Tota J. G. (1971). Effect of laser rays on wound healing. Am. J. Surg. 122, 532–535. 10.1016/0002-9610(71)90482-x 5098661

[B81] Mester E. Nagylucskay S. Döklen A. Tisza S. (1976). Laser stimulation of wound healing. Acta Chir. Acad. Sci. Hung 17, 49–55. 970061

[B82] Milares L. P. Assis L. Siqueira A. Claudino V. Domingos H. Almeida T. (2016). Effectiveness of an aquatic exercise program and low-level laser therapy on articular cartilage in an experimental model of osteoarthritis in rats. Connect. Tissue Res. 57, 398–407. 10.1080/03008207.2016.1193174 27220395

[B83] Mohammed N. Allam H. Elghoroury E. Zikri E. N. Helmy G. A. Elgendy A. (2018). Evaluation of serum beta-endorphin and substance P in knee osteoarthritis patients treated by laser acupuncture. J. Complement. Integr. Med. 15. 10.1515/jcim-2017-0010 29303777

[B84] Moon C. H. Kwon O. Woo C. H. Ahn H. D. Kwon Y. S. Park S. J. (2014). Therapeutic effect of irradiation of magnetic infrared laser on osteoarthritis rat model. Photochem Photobiol. 90, 1150–1159. 10.1111/php.12304 24962501

[B85] Mostafa M. Hamada H. A. Kadry A. M. Zahran S. S. Helmy N. A. (2022). Effect of high-power laser therapy Versus shock wave therapy on pain and function in knee osteoarthritis patients: a randomized controlled trial. Photobiomodul Photomed. Laser Surg. 40, 198–204. 10.1089/photob.2021.0136 34986012

[B86] Nambi G. (2021). Does low level laser therapy has effects on inflammatory biomarkers IL-1β, IL-6, TNF-α, and MMP-13 in osteoarthritis of rat models-a systemic review and meta-analysis. Lasers Med. Sci. 36, 475–484. 10.1007/s10103-020-03124-w 32833088

[B87] Nazari A. Moezy A. Nejati P. Mazaherinezhad A. (2019). Efficacy of high-intensity laser therapy in comparison with conventional physiotherapy and exercise therapy on pain and function of patients with knee osteoarthritis: a randomized controlled trial with 12-week follow up. Lasers Med. Sci. 34, 505–516. 10.1007/s10103-018-2624-4 30178432

[B88] Oliveira P. Santos A. A. Rodrigues T. Tim C. R. Pinto K. Z. Magri A. M. (2013). Effects of phototherapy on cartilage structure and inflammatory markers in an experimental model of osteoarthritis. J. Biomed. Opt. 18, 128004. 10.1117/1.Jbo.18.12.128004 24343447

[B89] Oliveira S. Andrade R. Hinckel B. B. Silva F. Espregueira-Mendes J. Carvalho O. (2021). *In vitro* and *in vivo* effects of light therapy on cartilage regeneration for knee osteoarthritis: a systematic review. Cartilage 13, 1700S–1719S. 10.1177/19476035211007902 33855869 PMC8804850

[B90] Oshima Y. Coutts R. D. Badlani N. M. Healey R. M. Kubo T. Amiel D. (2011). Effect of light-emitting diode (LED) therapy on the development of osteoarthritis (OA) in a rabbit model. Biomed. Pharmacother. 65, 224–229. 10.1016/j.biopha.2011.02.011 21658899

[B91] Ozcelik O. Cenk Haytac M. Kunin A. Seydaoglu G. (2008). Improved wound healing by low-level laser irradiation after gingivectomy operations: a controlled clinical pilot study. J. Clin. Periodontol. 35, 250–254. 10.1111/j.1600-051X.2007.01194.x 18269665

[B92] Page M. J. Green S. Kramer S. Johnston R. V. McBain B. Buchbinder R. (2014). Electrotherapy modalities for adhesive capsulitis (frozen shoulder). Cochrane Database Syst. Rev., CD011324. 10.1002/14651858.CD011324 25271097 PMC10898218

[B93] Page M. J. Green S. Mrocki M. A. Surace S. J. Deitch J. McBain B. (2016). Electrotherapy modalities for rotator cuff disease. Cochrane Database Syst. Rev. 2016, CD012225. 10.1002/14651858.CD012225 27283591 PMC8570637

[B94] Paolillo F. R. Paolillo A. R. João J. P. Frascá D. Duchêne M. João H. A. (2018). Ultrasound plus low-level laser therapy for knee osteoarthritis rehabilitation: a randomized, placebo-controlled trial. Rheumatol. Int. 38, 785–793. 10.1007/s00296-018-4000-x 29480363

[B95] Pearle A. D. Warren R. F. Rodeo S. A. (2005). Basic science of articular cartilage and osteoarthritis. Clin. Sports Med. 24, 1–12. 10.1016/j.csm.2004.08.007 15636773

[B96] Pinto N. C. de M. V. P. Ferreira N. L. Braga N. A. Aldred A. Gomes G. (2022). Customized photobiomodulation modulates pain and alters thermography pattern in patients with knee osteoarthritis: a randomized double-blind pilot study. Photobiomodul Photomed. Laser Surg. 40, 698–707. 10.1089/photob.2022.0067 36286574

[B97] Ramezani F. Razmgir M. Tanha K. Nasirinezhad F. Neshastehriz A. Bahrami-Ahmadi A. (2020). Photobiomodulation for spinal cord injury: a systematic review and meta-analysis. Physiol. Behav. 224, 112977. 10.1016/j.physbeh.2020.112977 32504695

[B98] Ranjbar R. Takhtfooladi M. A. (2016). The effects of photobiomodulation therapy on Staphylococcus aureus infected surgical wounds in diabetic rats. A microbiological, histopathological, and biomechanical study. Acta Cir. Bras. 31, 498–504. 10.1590/S0102-865020160080000001 27579876

[B99] Rankin I. A. Sargeant H. Rehman H. Gurusamy K. S. (2017). Low-level laser therapy for carpal tunnel syndrome. Cochrane Database Syst. Rev. 8, CD012765. 10.1002/14651858.CD012765 35611937 PMC6483673

[B100] Rayegani S. M. Raeissadat S. A. Heidari S. Moradi-Joo M. (2017). Safety and effectiveness of low-level laser therapy in patients with knee osteoarthritis: a systematic review and meta-analysis. J. Lasers Med. Sci. 8, S12–s19. 10.15171/jlms.2017.s3 29071029 PMC5642172

[B101] Regulski P. A. Szopinski K. T. Levičnik-Höfferle Š. (2023). Photobiomodulation therapy for the symptoms related to temporomandibular joint disk displacement. Case Rep. Dent. 2023, 5947168. 10.1155/2023/5947168 37089525 PMC10118889

[B102] Robbins S. R. Alfredo P. P. Junior W. S. Marques A. P. (2022). Low-level laser therapy and static stretching exercises for patients with knee osteoarthritis: a randomised controlled trial. Clin. Rehabil. 36, 204–213. 10.1177/02692155211047017 34714175

[B103] Roelandts R. (2002). The history of phototherapy: something new under the sun? J. Am. Acad. Dermatology 46, 926–930. 10.1067/mjd.2002.121354 12063493

[B104] Rosso M. P. de O. Buchaim D. V. Kawano N. Furlanette G. Pomini K. T. Buchaim R. L. (2018). Photobiomodulation therapy (PBMT) in peripheral nerve regeneration: a systematic review. Bioeng. (Basel) 5, 44. 10.3390/bioengineering5020044 29890728 PMC6027218

[B105] Samaan S. Sedhom M. G. Grace M. O. (2022). A randomized comparative study between high-intensity laser vs low-intensity pulsed ultrasound both combined with exercises for the treatment of knee osteoarthritis. Int. J. Rheum. Dis. 25, 877–886. 10.1111/1756-185x.14361 35678062

[B106] Sanches M. Assis L. Criniti C. Fernandes D. Tim C. Renno A. C. M. (2018). Chondroitin sulfate and glucosamine sulfate associated to photobiomodulation prevents degenerative morphological changes in an experimental model of osteoarthritis in rats. Lasers Med. Sci. 33, 549–557. 10.1007/s10103-017-2401-9 29196833

[B107] Shen X. Zhao L. Ding G. Tan M. Gao J. Wang L. (2009). Effect of combined laser acupuncture on knee osteoarthritis: a pilot study. Lasers Med. Sci. 24, 129–136. 10.1007/s10103-007-0536-9 18180980

[B108] Siriratna P. Ratanasutiranont C. Manissorn T. Santiniyom N. Chira-Adisai W. (2022). Short-term efficacy of high-intensity laser therapy in alleviating pain in patients with knee osteoarthritis: a single-blind randomised controlled trial. Pain Res. Manag. 2022, 1319165. 10.1155/2022/1319165 36313402 PMC9616657

[B109] Son J. Cho Y. Sung I. Kim I. Park B. Kim Y. (2014). Melatonin promotes osteoblast differentiation and mineralization of MC3T3-E1 cells under hypoxic conditions through activation of PKD/p38 pathways. J. Pineal Res. 57, 385–392. 10.1111/jpi.12177 25250639

[B110] Son J.-H. Park B.-S. Kim I.-R. Sung I.-Y. Cho Y.-C. Kim J.-S. (2017). A novel combination treatment to stimulate bone healing and regeneration under hypoxic conditions: photobiomodulation and melatonin. Lasers Med. Sci. 32, 533–541. 10.1007/s10103-017-2145-6 28091848

[B111] Song H. J. Seo H. J. Kim D. (2020). Effectiveness of high-intensity laser therapy in the management of patients with knee osteoarthritis: a systematic review and meta-analysis of randomized controlled trials. J. Back Musculoskelet. Rehabil. 33, 875–884. 10.3233/bmr-191738 32831189

[B112] Stancker T. G. Vieira S. S. Serra A. J. do Nascimento Lima R. Dos Santos Feliciano R. Silva J. A. (2018). Can photobiomodulation associated with implantation of mesenchymal adipose-derived stem cells attenuate the expression of MMPs and decrease degradation of type II collagen in an experimental model of osteoarthritis? Lasers Med. Sci. 33, 1073–1084. 10.1007/s10103-018-2466-0 29520686

[B113] Stausholm M. B. Naterstad I. F. Joensen J. Lopes-Martins Rá B. Sæbø H. Lund H. (2019). Efficacy of low-level laser therapy on pain and disability in knee osteoarthritis: systematic review and meta-analysis of randomised placebo-controlled trials. BMJ Open 9, e031142. 10.1136/bmjopen-2019-031142 31662383 PMC6830679

[B114] Stausholm M. B. Naterstad I. F. Alfredo P. P. Couppé C. Fersum K. V. Leal-Junior E. C. P. (2022). Short- and long-term effectiveness of low-level laser therapy combined with strength training in knee osteoarthritis: a randomized placebo-controlled trial. J. Clin. Med. 11, 3446. 10.3390/jcm11123446 35743513 PMC9225274

[B115] Steinmetz J. D. Culbreth G. T. Haile L. M. Rafferty Q. Lo J. Fukutaki K. G. (2023). Global, regional, and national burden of osteoarthritis, 1990–2020 and projections to 2050: a systematic analysis for the global burden of disease study 2021. Lancet Rheumatology 5, e508–e522. 10.1016/S2665-9913(23)00163-7 37675071 PMC10477960

[B116] Stelian J. Gil I. Habot B. Rosenthal M. Abramovici I. Kutok N. (1992). Improvement of pain and disability in elderly patients with degenerative osteoarthritis of the knee treated with narrow-band light therapy. J. Am. Geriatr. Soc. 40, 23–26. 10.1111/j.1532-5415.1992.tb01824.x 1727843

[B117] Su M. Nizamutdinov D. Liu H. Huang J. H. (2023). Recent mechanisms of neurodegeneration and photobiomodulation in the context of alzheimer’s disease. Int. J. Mol. Sci. 24, 9272. 10.3390/ijms24119272 37298224 PMC10253105

[B118] Tascioglu F. Armagan O. Tabak Y. Corapci I. Oner C. (2004). Low power laser treatment in patients with knee osteoarthritis. Swiss Med. Wkly. 134, 254–258. 10.4414/smw.2004.10518 15243853

[B119] Tim C. R. Martignago C. C. S. Assis L. Neves L. M. Andrade A. L. Silva N. C. (2022). Effects of photobiomodulation therapy in chondrocyte response by *in vitro* experiments and experimental model of osteoarthritis in the knee of rats. Lasers Med. Sci. 37, 1677–1686. 10.1007/s10103-021-03417-8 34554354

[B120] Tomazoni S. S. Leal-Junior E. C. Frigo L. Pallotta R. C. Teixeira S. de Almeida P. (2016). Isolated and combined effects of photobiomodulation therapy, topical nonsteroidal anti-inflammatory drugs, and physical activity in the treatment of osteoarthritis induced by papain. J. Biomed. Opt. 21, 108001. 10.1117/1.Jbo.21.10.108001 27752702

[B121] Tomazoni S. S. Leal-Junior E. C. Pallotta R. C. Teixeira S. de Almeida P. Lopes-Martins R. (2017). Effects of photobiomodulation therapy, pharmacological therapy, and physical exercise as single and/or combined treatment on the inflammatory response induced by experimental osteoarthritis. Lasers Med. Sci. 32, 101–108. 10.1007/s10103-016-2091-8 27726040

[B122] Trevisan E. S. Martignago C. C. S. Assis L. Tarocco J. C. Salman S. Dos Santos L. (2020). Effectiveness of led photobiomodulation therapy on treatment with knee osteoarthritis: a rat study. Am. J. Phys. Med. Rehabil. 99, 725–732. 10.1097/phm.0000000000001408 32167952

[B123] Tumilty S. Munn J. McDonough S. Hurley D. A. Basford J. R. Baxter G. D. (2010). Low level laser treatment of tendinopathy: a systematic review with meta-analysis. Photomed. Laser Surg. 28, 3–16. 10.1089/pho.2008.2470 19708800

[B124] Valter K. Tedford S. E. Eells J. T. Tedford C. E. (2024). Photobiomodulation use in ophthalmology - an overview of translational research from bench to bedside. Front. Ophthalmol. (Lausanne) 4, 1388602. 10.3389/fopht.2024.1388602 39211002 PMC11358123

[B125] Varongot-Reille C. Barrero-Santiago L. Cuenca-Martínez F. Paris-Alemany A. La Touche R. Herranz-Gómez A. (2023). Effectiveness of exercise on pain intensity and physical function in patients with knee and hip osteoarthritis: an umbrella and mapping review with meta-meta-analysis. Disabil. Rehabil., 1–15. 10.1080/09638288.2023.2252742 37697975

[B126] Vassão P. G. de Souza M. C. Silva B. A. Junqueira R. G. de Camargo M. R. Dourado V. Z. (2020). Photobiomodulation via a cluster device associated with a physical exercise program in the level of pain and muscle strength in middle-aged and older women with knee osteoarthritis: a randomized placebo-controlled trial. Lasers Med. Sci. 35, 139–148. 10.1007/s10103-019-02807-3 31144070

[B127] Vassão P. G. de Souza A. C. F. da Silveira Campos R. M. Garcia L. A. Tucci H. T. Renno A. C. M. (2021a). Effects of photobiomodulation and a physical exercise program on the expression of inflammatory and cartilage degradation biomarkers and functional capacity in women with knee osteoarthritis: a randomized blinded study. Adv. Rheumatol. 61, 62. 10.1186/s42358-021-00220-5 34656170

[B128] Vassão P. G. Parisi J. Penha T. F. C. Balão A. B. Renno A. C. M. Avila M. A. (2021b). Association of photobiomodulation therapy (PBMT) and exercises programs in pain and functional capacity of patients with knee osteoarthritis (KOA): a systematic review of randomized trials. Lasers Med. Sci. 36, 1341–1353. 10.1007/s10103-020-03223-8 33392780

[B129] Wang P. Liu C. Yang X. Zhou Y. Wei X. Ji Q. (2014). Effects of low-level laser therapy on joint pain, synovitis, anabolic, and catabolic factors in a progressive osteoarthritis rabbit model. Lasers Med. Sci. 29, 1875–1885. 10.1007/s10103-014-1600-x 24890034

[B130] Wang Y. Huang Y.-Y. Wang Y. Lyu P. Hamblin M. R. (2017). Photobiomodulation of human adipose-derived stem cells using 810nm and 980nm lasers operates via different mechanisms of action. Biochimica Biophysica Acta. General Subj. 1861, 441–449. 10.1016/j.bbagen.2016.10.008 27751953 PMC5195895

[B131] Wang W. Niu Y. Jia Q. (2022). Physical therapy as a promising treatment for osteoarthritis: a narrative review. Front. Physiol. 13, 1011407. 10.3389/fphys.2022.1011407 36311234 PMC9614272

[B132] Whittaker P. (2004). Laser acupuncture: past, present, and future. Laser. Med. Sci. 19, 69–80. 10.1007/s10103-004-0296-8 15349795

[B133] Wu F. Zhang R. Shen X. Lao L. (2014). Preliminary study on pain reduction of monosodium iodoacetate-induced knee osteoarthritis in rats by carbon dioxide laser moxibustion. Evid. Based Complement. Altern. Med. 2014, 754304. 10.1155/2014/754304 25013448 PMC4074960

[B134] Wu M. Luan L. Pranata A. Witchalls J. Adams R. Bousie J. (2022). Is high intensity laser therapy more effective than other physical therapy modalities for treating knee osteoarthritis? A systematic review and network meta-analysis. Front. Med. (Lausanne) 9, 956188. 10.3389/fmed.2022.956188 36186780 PMC9520262

[B135] Wyszyńska J. Bal-Bocheńska M. (2018). Efficacy of high-intensity laser therapy in treating knee osteoarthritis: a first systematic review. Photomed. Laser Surg. 36, 343–353. 10.1089/pho.2017.4425 29688827

[B136] Yamada E. F. Bobinski F. Martins D. F. Palandi J. Folmer V. da Silva M. D. (2020). Photobiomodulation therapy in knee osteoarthritis reduces oxidative stress and inflammatory cytokines in rats. J. Biophot. 13, e201900204. 10.1002/jbio.201900204 31568634

[B137] Yamada E. F. Dos Santos Stein C. Moresco R. N. Bobinski F. Palandi J. Fernandes P. F. (2022). Photobiomodulation and Sida tuberculata combination declines the inflammation’s markers in knee-induced osteoarthritis. Lasers Med. Sci. 37, 193–204. 10.1007/s10103-020-03207-8 33417067

[B138] Youssef E. F. Muaidi Q. I. Shanb A. A. (2016). Effect of laser therapy on chronic osteoarthritis of the knee in older subjects. J. Lasers Med. Sci. 7, 112–119. 10.15171/jlms.2016.19 27330707 PMC4909009

[B139] Ys L. Yj C. Cl L. Fy K. Yh T. Ch C. (2023). Effects of photobiomodulation as an adjunctive treatment in chronic obstructive pulmonary disease: a narrative review. Lasers Medical Science 38. 10.1007/s10103-022-03661-6 PMC9883131 36707463

[B140] Yurtkuran M. Alp A. Konur S. Ozçakir S. Bingol U. (2007). Laser acupuncture in knee osteoarthritis: a double-blind, randomized controlled study. Photomed. Laser Surg. 25, 14–20. 10.1089/pho.2006.1093 17352632

[B141] Zamani A. R. N. Saberianpour S. Geranmayeh M. H. Bani F. Haghighi L. Rahbarghazi R. (2020). Modulatory effect of photobiomodulation on stem cell epigenetic memory: a highlight on differentiation capacity. Lasers Med. Sci. 35, 299–306. 10.1007/s10103-019-02873-7 31494789

[B142] Zhao L. Shen X. Cheng K. Deng H. Ding G. Tan M. (2010). Validating a nonacupoint sham control for laser treatment of knee osteoarthritis. Photomed. Laser Surg. 28, 351–356. 10.1089/pho.2009.2511 19860569

[B143] Zhao L. Cheng K. Wu F. Du J. Chen Y. Tan M. T. (2021). Effect of laser moxibustion for knee osteoarthritis: a multisite, double-blind randomized controlled trial. J. Rheumatol. 48, 924–932. 10.3899/jrheum.200217 32611673

