# Supplementary material for: Photobiomodulation for the treatment of knee osteoarthritis: therapeutic effects and molecular mechanism
Source: Front Cell Dev Biol. 2026 Mar 2;14:1744761. doi: 10.3389/fcell.2026.1744761 (PMC12989597; doi:10.3389/fcell.2026.1744761)
Supplement: Supplementary file 1 [file Table1.docx]

**Table 1 Clinical evidences of PBM therapy for KOA patients**

| **Reference** | **Study design** | **Device** | **Combined treatment** | **Control** | **Parameters** | **Duration of treatment** | **Outcome measure** | **Results** | **Quality(PEDro)** |
| --- | --- | --- | --- | --- | --- | --- | --- | --- | --- |
| Stelian,et al(Stelian et al., 1992) | RCT | LLLT | No | Placebo | λ(nm): 633/830  P(mW):18/75/25/270  Beam area (cm^2^): 2  T(min):15  ED (J/cm^2^): 5.1/5.6  E(J):10.3,11.1  PD(mW/cm^2^ ):8/34/11/122 | 2/day,  10 days | SF-MPQ, PPI, VAS, DIQ | Reduce pain and improve disability | Moderate |
| Hegedus,et al(Hegedus et al., 2009) | RCT | LLLT | No | Placebo | λ(nm):830  P(mW):50/0.5  Beam area(mm^2^):0.5  T(min):N/A  ED (J/cm^2^):48  E(J): 6/point  PD(W/cm^2^):10 | 2/week,  4 weeks | Thermography, joint flexion, circumference, pressure sensitivity,VAS | Reduce pain and miccirculation | High |
| Jankaew, et al(Jankaew et al., 2023) | RCT | LLLT | No | Sham LED | λ(nm):808/660  P(mW): 300  Beam area(cm^2^): N/A  T(min):15  ED(J/cm^2^): N/A  E(J):5.76J  PD(mW/cm^2^): N/A | 3/week,  8 weeks | muscle strength, sit-to-stand test, 40 m fast‑paced walk, stair climbing, TUG | Improve muscle strength and function | High |
| Fukuda, et al(Fukuda et al., 2011) | RCT | LLLT | No | Placebo | λ(nm):904  P(mW):60  Beam area(cm^2^): 0.5  T(min): 4.5min  ED (J/cm^2^): N/A  E(J):3/point, total 27  PD(mW/cm^2^ ): N/A  f(Hz):700 | 3/week,  3 weeks | Lequesne index, VNPS, TUG, ROM, muscle strength | Reduce pain and improve function | Moderate-high |
| S.Gopal, et al(S et al., 2017) | RCT | LLLT | No | Placebo | λ(nm):905  P(mW): 25  Beam area(cm^2^):1  T(min):1/point, total 8  ED (J/cm^2^):N/A  E(J): 1.5/point, total 12  PD(mW/cm^2^ ) :N/A | 3/week  4 weeks | VAS, radiographic analysis, biochemical analysis | Reduce pain and increase cartilage thickness | Moderate-high |
| Rayegani, et al(Rayegani et al., 2017) | SR | LLLT | No | Placebo | N/A | N/A | VAS, WOMAC | Reduce pain and improve function | N/A |
| Stausholm, et al(Stausholm et al., 2019) | SR | LLLT | No | Placebo | N/A | N/A | VAS,WOMAC | Reduce pain and improve function | N/A |
| Bülow,et al(Bülow et al., 1994) | RCT | LLLT | No | Placebo | λ(nm):830  P(mW):25  Beam area (cm^2^): 0.28  T(min):15  ED (J/cm^2^):N/A  E(J):22.5  PD(mW/cm^2^ ):N/A | 2-4/week,  3 weeks,  9 sessions | Medicine requirements, pain and palpation tenderness assessment | Ineffective | Moderate |
| Tascioglu,et al(Tascioglu et al., 2004) | RCT | LLLT | No | Placebo | λ(nm):830  P(mW): 50  Beam area (cm^2^): 0.01  T(min): 5  ED (J/cm^2^):N/A  E(J):3/1.5/point, total 15/7.5  PD(mW/cm^2^ ):N/A | 5/week,  2 weeks | WOMAC | Ineffective | Moderate |
| Huang, et al(Huang et al., 2015) | SR | LLLT | No | Placebo | λ(nm):830,850,904  P(mW): 4-800  Beam area(cm^2^):N/A  T(min): N/A  ED (J/cm^2^):0.76-50  E(J): 0.48-12/point  PD(mW/cm^2^):N/A | 2-5/week,  8-20 sessions | VAS, WOMAC | Ineffective | High |
| Yurtkuran,et al(Yurtkuran et al., 2007) | RCT | Laser acupuncture | No | Placebo | λ(nm):904  P(mW):4  Beam area(cm^2^):0.4  T(min):20  ED (J/cm^2^):N/A  E(J):0.48/session  PD(mW/cm^2^ ):10 | 1/day,  10 days | VAS, 50 foot walking time, KC,MTS, WOMAC,NHP | Reduce periarticular swelling | Moderate |
| Al Rashoud, et al(Al Rashoud et al., 2014) | RCT | Laser acupuncture | No | Placebo | λ(nm):830  P(mW):30  Beam area(cm^2^):0.28  T(s):40/point, total 200  ED (J/cm^2^):4  E(J):1.2/point, total 6  PD(mW/cm^2^ ): N/A | 9 sessions | VAS,SKFS | Reduce pain and improve function | Moderate |
| Helianthi, et al(Helianthi et al., 2016) | RCT | Laser acupuncture | No | Placebo | λ(nm):785  P(mW): 50  Beam area(cm^2^): N/A  T(s):80/point,total 400  ED (J/cm^2^): N/A  E(J): 4/point, total 20  PD(mW/cm^2^ ):25 | 2/week  5 weeks | VAS, Lequesne index | Reduce pain | Moderate |
| Mohammed, et al(Mohammed et al., 2018) | RCT | Laser acupuncture | No | Placebo | λ(nm):808  P(mW): 90  Beam diameter(mm):2  T(min): 1/point, total 5  ED (J/cm^2^):N/A  E(J): 70.2  PD(mW/cm^2^ ) :N/A | 3/week  4 weeks | VAS, radiological imaging, laboratory test | Reduce pain, increase serum beta-endorphin and decrease substance P | Moderate |
| Liao, et al(Liao et al., 2020) | RCT | Laser acupuncture | No | Placebo | λ(nm):780/830  P(mW): 50/30  Beam area(cm^2^): N/A  T(min):15  ED (J/cm^2^): N/A  E(J): 216  PD(mW/cm^2^):N/A | 3/week,  4 weeks | VAS,PPT  Lequesne index | Reduce pain and improve function | Moderate |
| Shen, et al(Shen et al., 2009) | RCT | Laser acupuncture | No | Placebo | λ(nm):650/10600  P(mW):36/200  Beam diameter(mm):2  T(min):20  ED (J/cm^2^): N/A  E(J): N/A  PD(mW/cm^2^ ): N/A | 1/other day,  3/week,  4 weeks | WOMAC | Reduce pain | Moderate |
| Zhao, et al(Zhao et al., 2010) | RCT | Laser acupuncture | No | Placebo | λ(nm):650/10600  P(mW):36/200  Beam diameter(mm):2  T(min):20  ED (J/cm^2^): N/A  E(J):43.2/120  PD(mW/cm^2^ ): N/A  f(Hz):40 | 3/week  4 weeks | WOMAC | Reduce pain | Moderate |
| Lin, et al(Lin et al., 2020) | RCT | Laser moxibustion | No | Placebo | λ(nm):10600  P(mW): 160-180  Beam diameter(cm): 2  T(min):20  ED(J/cm^2^): 61.2-68.8  E(J):N/A  PD(mW/cm^2^):N/A | 3/week,  4 weeks | WOMAC, SF-36 | Reduce pain and improve function | Moderate |
| Zhao, et al(Zhao et al., 2021) | RCT | Laser moxibustion | No | Placebo | λ(nm):1064  P(mW): 160-180  Beam area(cm^2^): N/A  T(min):20  ED (J/cm^2^):61.2-68.8  E:N/A  PD(mW/cm^2^): N/A | 3/week,  4 weeks | VAS,WOMAC | Reduce pain and improve function | Moderate |
| Chen, et al(Chen et al., 2019) | SR | Laser acupuncture | No | Placebo | NA | NA | VAS,WOMAC | Reduce pain at short term | N/A |
| de Matos Brunelli  Braghin, et al(de Matos Brunelli Braghin et al., 2019) | RCT | LLLT | Exercise | Control,  Exercise | λ(nm):808  P(mW): 100  Beam area(cm^2^):0.028  T(s):56/point, total 560  ED (J/cm^2^):200  E(J): 5.6/point, total 56  PD(mW/cm^2^):N/A | 2/week,  2 months,  15 sessions | WOMAC,  gait evaluation | LLLT+exercise significantly reduce pain , improve function and gait variables | High |
| Kholvadia, et al(Kholvadia et al., 2019) | RCT | LLLT | Exercise | Exercise | λ(nm): N/A  P(mW): N/A  Beam area(cm^2^):N/A  T(min):35-45  ED (J/cm^2^): N/A  E(J): N/A  PD(mW/cm^2^):N/A | 2/week,  12 sessions | WOMAC, ROM, sit–to-stand test, knee circumference | Reduce pain and improve function，LLLT plus exercise is the best | High |
| Robbins, et al(Robbins et al., 2022) | RCT | LLLT | Exercise | Control,  Exercise,  Exercise+Placebo | λ(nm):904  P(mW): 40  Beam area(cm^2^):0.1  T(min):N/A  ED(J/cm^2^): N/A  E(J):3/point, total 27  PD(mW/cm^2^): N/A  f(Hz):9500 | 3/week  3 weeks | VAS, ,WOMAC, KOOS, Lequesne index, medication intake, ROM,TUG | LLLT combined with stretching exercises reduce pain, improve activities of daily living, stiffness, muscle shortening and range of motion | High |
| Gur,et al(Gur et al., 2003) | RCT | LLLT | Exercise | Placebo+Exercise | λ(nm):904  P(mW):10/11.2  Beam area (cm^2^): 1  T(min): 5/3min  ED (J/cm^2^):N/A  E(J):3/2  PD(mW/cm^2^ ):N/A  f(kHz):2.5/2.8 | 2 weeks,  10 sessions | VAS, ROM, WOMAC,QOL | Reduce pain, improve function and QOL | High |
| Alfredo, et al(Alfredo et al., 2012) | RCT | LLLT | Exercise | Placebo+Exercise | λ(nm):904  P(mW):60  Beam area(cm^2^): 0.5  T(s):50/point  ED (J/cm^2^):N/A  E(J):3/point, total 27  PD(mW/cm^2^ ): N/A  f(Hz):700 | 1/day,  5 days/week,  2 weeks | Lequesne index, VAS, WOMAC, ROM, muscle strength | Reduce pain, improve function and activity | High |
| Alghadir, et al(Alghadir et al., 2014) | RCT | LLLT | Exercise | Placebo+Exercise | λ(nm):850  P(mW):100  Beam area(cm^2^):0.01  T(min):8  ED (J/cm^2^):48  E(J):6/point, total 48  PD(mW/cm^2^ ): N/A | 2/week,  4 weeks | VAS, WOMAC, ambulation duration | Reduce pain and improve function | High |
| Youssef, et al(Youssef et al., 2016) | RCT | LLLT | Exercise | Placebo+Exercise | λ(nm):880/904  P(mW): 50,60  Beam area(cm^2^): 0.5  T(s):60/50/point, total 480/450  ED (J/cm^2^):6/3  E(J): 6,3/point, total 48,27  PD(mW/cm^2^ ) :N/A  f(Hz): 700 | 2/week  8 weeks | VAS, WOMAC,ROM, muscle strength | Reduce pain and improve function | High |
| Alfredo, et al(Alfredo et al., 2018) | RCT | LLLT | Exercise | Placebo+ Exercise | λ(nm):904  P(mW): N/A  Beam area(cm^2^): N/A  T(min): N/A  ED (J/cm^2^):N/A  E(J): 3/point  PD(mW/cm^2^ ) :N/A | 2/week  3 weeks | VAS, WOMAC, Lequesne index, paracetamol consumption | Pain and function post-intervention improvements from LLLT plus strengthening exercises were maintained for six months. | High |
| Alfredo, et al(Alfredo et al., 2022) | RCT | LLLT | Exercise | Placebo | λ(nm):904  P(mW): 60  Beam area(cm^2^):0.5  T(s):50/point,total 450  ED (J/cm^2^): N/A  E(J):3/point, total 27  PD(mW/cm^2^): N/A  f(Hz):700 | 3/week,  8 weeks | WOMAC, TUG, Lequesne index, ROM, muscle strength, medication intake relief | Reduce pain, improve function and intake of medication | High |
| Stausholm, et al(Stausholm et al., 2022) | RCT | LLLT | Strength training | Placebo+strength training | λ(nm):904  P(mW):60  Beam area(cm^2^): N/A  T(s):50, total 750  ED(J/cm^2^): N/A  E:3J/point, total 45  PD(mW/cm^2^): N/A | 3/week,  3 weeks | VAS,KOOS,PPT, ROM, ultrasonography, sit-to-stand test | Reduce pain, LLLT provide a positive add-on effect in terms of reduced pain medication usage and increased performance in the sit-to-stand test | High |
| Vassão,et al(Vassão et al., 2020) | RCT | LLLT | Exercise | Placebo  +Exercise,  Exercise | λ(nm):808  P(mW): 100  Beam area(cm^2^): 0.05  T(s): 40  ED (J/cm^2^): 91  E(J): 4/point, total 56  PD(W/cm^2^):2 | 2/week,  8 weeks | NRS, 6MWT,TUG, MVIT | LLLT did not have any extra effect along with the effects of exercise in improving the distance walked, the TUG, and the muscle strength | High |
| Vassão,et al(Vassão et al., 2021a) | RCT | LLLT | Exercise | Control | λ(nm):808  P(mW): 100  Beam area(cm^2^): 0.05  T(s): 40  ED (J/cm^2^): 91  E(J): 4/point, total 56  PD(W/cm^2^):2 | 2/week,  8 weeks | WOMAC,ELISA | Increase IL-10 levels, not promote effect of exercise in function | High |
| Vassão,et al(Vassão et al., 2021b) | SR | PBM | Exercise | No | N/A | N/A | WOMAC,VAS,NRS | Cotroversy | N/A |
| Malik, et al(Malik et al., 2023) | SR | LLLT | Exercise | Placebo+Exercise | λ(nm):640-905  P(mW): N/A  Beam area(cm^2^): N/A  T(min): N/A  ED(J/cm^2^): N/A  E(J):4-8J/point  PD(mW/cm^2^): N/A | 2/week,  10-16 sessions | VAS, WOMAC, ROM, muscle strength | LLLT plus exercise is no more effective than placebo LLLT plus exercise in improving ROM, muscle strength, and function | N/A |
| Melo Mde,  et al(Melo Mde et al., 2015) | RCT | LLLT | NES | NES | λ(nm):810  P(mW): 200  Beam area(cm^2^):0.0364  T(s): 30/20/point, total 180/120  ED (J/cm^2^):0.218  E(J): 4/6/point, total 24/36  PD(mW/cm^2^):N/A | 2/week,  8 weeks | VAS, 6MW ,TUG, ultrasonography | Reduce pain and improve function, no significant differences compared to NES | High |
| de Oliveira Melo, et al(de Oliveira Melo et al., 2016) | RCT | LLLT | NES | NES | λ(nm):810  P(mW): 200  Beam area(cm^2^):0.0364  T(min):30/20/point, total 180/120  ED (J/cm^2^):0.218  E(J): 4/6/point, total 24/36  PD(mW/cm^2^):N/A | 2/week,  8 weeks | WOMAC,ultrasonography, muscle strength, electromyography | Increases health status and electrical activity, no effect on muscle mass. | High |
| Fakhari, et al(Fakhari et al., 2021) | RCT | LLLT | No | Ozone | λ(nm):980  P(mW): 100  Beam area(cm^2^):0.5  T(min):2.5/point, total 17.5  ED (J/cm^2^):  E(J):6/point, total 42  PD(mW/cm^2^):N/A | 3/week,  4 weeks | VAS, WOMAC | Reduce pain and improve function, ozone is more effective | High |
| Alqualo-Costa, et al(Alqualo-Costa et al., 2021) | RCT | LLLT | No | Placebo | λ(nm):904  P(mW): 40  Beam area(cm^2^):0.5  T(min):1.25/point, total 11.25  ED (J/cm^2^): N/A  E(J):3/point, total 27  PD(mW/cm^2^): N/A  f(Hz):9500 | 3/week  4 weeks | NRS,WOMAC,Lequesne index, TUG | Reduce pain | High |
| Elboim-Gabyzon, et al(Elboim-Gabyzon and Nahhas, 2023) | RCT | LLLT | No | PEMFT | λ(nm): N/A  P(mW): N/A  Beam area(cm^2^): 20  T(min):3/point, total 15  ED(J/cm^2^): 8  E(J): N/A  PD(mW/cm^2^): N/A  f(Hz):2 | 2/week,  3 weeks | VAS,WOMAC,TUG,10MW | Reduce pain and improve function, PEMFT is more effective | High |
| Paolillo, et al(Paolillo et al., 2018) | RCT | LLLT | US, Exercise | Placebo | λ(nm):808  P(mW): 100  Beam area(cm^2^):3.5  T(min): 3/region, total 15  ED (J/cm^2^):7/laser diode, 28/region, total 142  E(J): 18/laser diode, 72/region,  total 360  PD(W/cm^2^):1  f(MHz):1 | 1/week  3 months | PPT, sit-to-stand test | Reduce pain and improve function | High |
| Angelova, et al(Angelova and Ilieva, 2016) | RCT | HILT | No | Placebo | λ(nm):1064  P(mW): 12000  Beam area(cm^2^):25  T(min):2/10  ED (J/cm^2^):12/120  E(J): total 300/3000 PD(mW/cm^2^):N/A  f(Hz):25 | 1/day,  7 sessions | VAS, dolorimetry, gait analysis | Reduce pain and improve function | Moderate-high |
| Akaltun, et al(Akaltun et al., 2021) | RCT | HILT | Exercise | Placebo+Exercise | λ(nm):1064  P(mW): 12000  Beam area(cm^2^):25  T(min): N/A  ED (J/cm^2^):120  E:3000  PD(mW/cm^2^):N/A  f(Hz):25 | 5/week,  2 weeks | WOMAC, VAS， Ultrasonographic | Reduce pain, improve function, increase femoral cartilage thickness and ROM | High |
| Wyszyńska, et al(Wyszyńska and Bal-Bocheńska, 2018) | SR | HILT | No | Placebo/ Comparision | N/A | N/A | VAS,WOMAC | Reduce pain and improve function | N/A |
| Song, et al(Song et al., 2020) | SR | HILT | No | Control | N/A | N/A | WOMAC,VAS | Reduce pain and WOMAC stiffness | N/A |
| Gworys, et al(Gworys et al., 2012) | RCT | LLLT,  HILT | No | Placebo | λ(nm):810(LLLT),  808/905(HILT)  P(mW):400/1100  Beam area(cm^2^):N/A  T(min):N/A  ED (J/cm^2^):12.7/6.21/3.28  E(J):8/12.4/6.6/point, total 96/99.2/79.2  PD(mW/cm^2^ ):634.9  f(Hz):2000 | 1/day,  5days/week  2 weeks | Lequesne index, VAS, modified Laitinen questionnaire | Reduce pain and improve function | Moderate-high |
| Kheshie,et al(Kheshie et al., 2014) | RCT | HILT,  LLLT | Exercise | Placebo+Exercise | λ(nm):830(LLLT)  P(mW): 800(LLLT)  Beam area(cm^2^):N/A  T(min):32  ED (J/cm^2^):50  E(J): 1250  PD(mW/cm^2^ ): N/A  f(Hz):1000 | 2/week  6 weeks | VAS, WOMAC | Reduce pain and improve function | Moderate-high |
| Ahmad, et al(Ahmad et al., 2022) | SR | LLLT,  HILT | Exercise | No | N/A | N/A | WOMAC,VAS | Reduce pain and improve function , HILT is more effective | N/A |
| Mostafa, et al(Mostafa et al., 2022) | RCT | HILT | CPT | ESWT+CPT | λ(nm):NA  P(mW):NA  Beam area(cm^2^):N/A  T(min):N/A  ED(J/cm^2^): 1.5  E(J): N/A  PD(mW/cm^2^): N/A  f(Hz):30 | 3/week  4 weeks | VAS,WOMAC, 6MWT | Reduce pain and improve function , HILT is more effective | Moderate-high |
| Samaan, et al(Samaan et al., 2022) | RCT | HILT | Exercise | Control, LIPUS+Exercise | λ(nm):1064  P(mW):12000  Beam area(cm^2^):N/A  T(min):N/A  ED(J/cm^2^): 120  E: 3000  PD(mW/cm^2^): N/A  f(Hz):25 | 5/week  2 weeks | VAS,WOMAC,ROM | Reduce pain and improve function , HILT+exercise is more effective | Moderate-high |
| Nazari, et al(Nazari et al., 2019) | RCT | HILT | No | Exercise,  CPT | λ(nm):1064  P(mW): 5000  Beam area(cm^2^): N/A  T(min):8  ED (J/cm^2^): 60  E(J): 2400  PD(mW/cm^2^):N/A  f(Hz):30 | 3/week,  4 weeks | VAS,ROM,TUG,6MWT,WOMAC | Reduce pain, improve function and stiffness | Moderate-high |
| Wu, et al(Wu et al., 2022) | SR | HILT | No | Other physical therapy modalities | N/A | N/A | WOMAC,VAS | Reduce pain and improve function , HILT is more effective | N/A |
| Kim, et al(Kim et al., 2016) | RCT | HILT | CPT | CPT | λ(nm):Unknown  P(mW): N/A  Beam area(cm^2^): N/A  T(m):10  E(mJ/cm^2^):1500  E(J): N/A  PD(mW/cm^2^ ) :N/A  f(Hz): 11 | 3/week  4 weeks | VAS,WOMAC | Reduce pain and improve function | Moderate-high |
| Siriratna, et al(Siriratna et al., 2022) | RCT | HILT | No | Control | λ(nm):808/905  P(mW): 25000  Beam area(cm^2^):3.14  T(min):8  ED(J/cm^2^):22.39  E:70.31J/point, total 562.5  PD(mW/cm^2^): N/A  f(Hz):2000 | 2-3/week,  4-5 weeks,  10 sessions | VAS,WOMAC | Reduce pain | Moderate-high |
| Ammendolia, et al(Ammendolia et al., 2021) | RCT | HILT | GS | HILT+Placebo | λ(nm):905  P(mW): 4500  Beam area(cm^2^):N/A  T(min): 20  ED (J/cm^2^):70  E: N/A  PD(mW/cm^2^):N/A | 3/week,  4 weeks | VAS | Reduce pain, HILT+GS is more effective | Moderate-high |
| Alayat, et al(Alayat et al., 2017) | RCT | HILT | GS, Exercise | GS+Exercise,  Placebo+ Exercise | λ(nm):1064  P(mW): 10500  Beam area(cm^2^):0.2  T(min):7  ED (J/cm^2^):15  E: 3000  PD(mW/cm^2^ ) :N/A  f(Hz):10-30 | 2/week  6 weeks | VAS, WOMAC, ultrasonography | Reduce pain and improve function | Moderate-high |
| Hsieh, et al(Hsieh et al., 2012) | RCT | LED | No | Placebo | λ(nm):890  P(mW): 6240  Beam area(cm^2^):N/A  T(min): 40  ED (J/cm^2^): 2.08/min, total 83.2  E(J):2.08 /cm^2^/min, total 83.2/cm^2^  PD(mW/cm^2^ ): N/A | 3/week  2 weeks | KOOS, Lysholm Knee Scale, HADS, MFI, Chronic Pain Grade questionnaire | Short-term LED therapy provided no beneficial effects to body functions, activities, participation, and quality of life | Moderate-high |
| Ammar, et al(Ammar, 2014) | RCT | LED,  LLLT | Exercise | No | λ(nm):890(LED)/  850(LLLT)  P(mW): 100(LLLT)  Beam area(cm^2^):0.01(LLLT)  T(min):30(LED)/10(LLLT)  ED (J/cm^2^):up to 1.6/min  E(J): 5/point(LLLT),  62.4J/cm^2^ (LED)  PD(mW/cm^2^):up to 10 | 2/week  6 weeks | VAS, WOMAC, LEFS | Reduce pain and improve function , no differeces between two groups | Moderate-high |
| de Paula Gomes, et al(de Paula Gomes et al., 2018) | RCT | LLLT,  LED | Exercise | Placebo+Exercise | λ(nm):905/640/875  P(mW):0.9/15/17.5  Beam area(cm^2^): 0.44/0.9  T(min):1/quadrant, total 60  ED (J/cm^2^): 0.12/1/1.17  E(J):7.85/quadrant, total 23.55  PD(mW/cm^2^):2.25/16.66/19.44 | 2/week  5 weeks | NRS, WOMAC, LEFS, FRT | Reduce pain | Moderate-high |
| Pinto, et al(Pinto et al., 2022) | RCT | LED | No | Placebo | λ(nm):850  P(mW): N/A  Beam area(cm^2^):N/A  T(min):5-8  ED(J/cm^2^):18-48  E(J):5.3-14/point, total 526-1402  PD(mW/cm^2^): 60-100 | 2/week,  5 weeks | NRS,QOL | Reduce pain and QOL | Moderate-high |

RCT: Randomized Controlled Trial; CCT: Controlled Clinical Trial; DS: Descriptive Study; SR: Systematic Review; PBM: Photobiomodulation; LLLT: Low-Level Laser Therapy; HILT: High-Level Laser Therapy; LED: Light Emitting Diodes; ED: Energy Density; PD: Power Density; SF-MPQ: Short-Form Mcgill Pain Questionnaire; VAS: Visual Analogue Scale; ROM: Range Of Motion; DIQ: Disability Index Questionnaire; WOMAC: Western Ontario And Mcmaster University Osteoarthritis Index; KC: Knee Circumference; MTS: Medial Tenderness Score; NHP: Nottingham Health Profile; VNPS: Visual Numerical Pain Scale; TUG: Timed Up and Go Test; KOOS: Knee Injury and Osteoarthritis Outcome Score; HADS: Hospital Anxiety And Depression Scale; MFI:Multidimensional Fatigue Inventory; SKFS:Saudi Knee Function Scale; LEFS : Lower Extremity Functional Scale; 6MWT :6-min Walk Test; ELISA : Enzyme-Linked Immunosorbent Assay; K-L: Kellgren-Lawrence; FRT :Functional Reach Test; NRPS: Numerical Rating Pain Scale;PPT: Pressure Pain Threshold; FRT :Functional Reach Test ; SF-36: Short Form 36; MVIT :Maximal Voluntary Isometric Torque; 10 MW: 10-min Walk Test; QOL: Quality of Life; LIPUS: Low Intensity Pulsed Ultrasound; GS: Glucosamine Sulfate; CPT: Conservative Physical Therapy; PEMFT: Pulsed Electromagnetic Field Therapy; ESWT: Extracorporeal Shock Wave Therapy; US: Ultrasound
